# The Perils of Being Populous: Control and Conservation of Abundant Kangaroo Species

**DOI:** 10.3390/ani11061753

**Published:** 2021-06-11

**Authors:** David Benjamin Croft, Ingrid Witte

**Affiliations:** 1 School of Biological Earth & Environmental Sciences, UNSW Sydney, Sydney, NSW 2052, Australia; 2 Rooseach@Rootourism^TM^, Adelaide River, NT 0846, Australia; roosearch@protonmail.com

**Keywords:** kangaroo, wallaroo, wildlife management, human–wildlife conflict, abundance, consumptive use, aboriginal use, fire

## Abstract

**Simple Summary:**

Kangaroos likely prospered for most of the last 65,000 years under the landscape management of Australia’s first people. From the arrival of British colonists in 1788, European agricultural practices, crops and livestock transformed the landscape to one less favourable to indigenous flora and fauna. However, the six species of large kangaroos persisted and came into conflict with cropping and pastoral enterprises, leading to controls on their abundance. After mass killing for bounties, a commercial industry emerged in the 1970s to sell meat and hides into domestic and international markets. The further intention was to constrain kangaroo abundance while sustaining kangaroos in the landscape. Human–human conflict has emerged about the necessity and means of this lethal control. Further control of the abundance of four of the six species is promoted. Their abundance is considered by some as a threat to biodiversity in conservation reserves, removing these as a haven. We therefore propose returning the kangaroos’ stewardship to the current and future generations of Aboriginal Australians. We envisage that a marriage of localised consumptive (bush tucker) and non-consumptive (wildlife tourism) uses in the indigenous-protected-area estate can better sustain abundant kangaroo populations into the future.

**Abstract:**

Australia’s first people managed landscapes for kangaroo species as important elements of their diet, accoutrements and ceremony. This developed and persisted for about 65,000 years. The second wave of colonists from the United Kingdom, Ireland and many subsequent countries introduced familiar domesticated livestock and they have imposed their agricultural practices on the same landscapes since 1788. This heralded an ongoing era of management of kangaroos that are perceived as competitors to livestock and unwanted consumers of crops. Even so, a kangaroo image remains the iconic identifier of Australia. Kangaroo management is shrouded in dogma and propaganda and creates a tension along a loose rural–city divide. This divide is further dissected by the promotion of the consumption of kangaroo products as an ecological good marred by valid concerns about hygiene and animal welfare. In the last decade, the fervour to suppress and micro-manage populations of some kangaroo species has mounted. This includes suppression within protected areas that have generally been considered as safe havens. This review explores these tensions between the conservation of iconic and yet abundant wildlife, and conflict with people and the various interfaces at which they meet kangaroos.

## 1. Introduction

The most diverse family of marsupials (Marsupialia) is the Macropodidae (~67 species with four recent extinctions), which includes small to medium-sized browsing and grazing herbivores, such as the pademelons (*Thylogale*), hare-wallabies (*Lagorchestes*, *Lagostrophus*), nailtail wallabies (*Onychogalea*), rock-wallabies (*Petrogale*), brush wallabies (*Setonix*, *Wallabia*, *Notamacropus*), kangaroos (*Macropus, Osphranter*) and tree-kangaroos (*Dendrolagus*). Most are found only in Australia, but New Guinea contributes a higher diversity of tree-kangaroos (~12 species) than Australia (2 species) and two endemic genera of forest wallabies (*Dorcopsis* and *Dorcopsulus*) [1]. Modern humans, *Homo sapiens*, walked out of Africa as early as 220,000 years ago [2] or at least within a range from 126–74 thousand years ago [3]. These early humans reached Australia (Sahul) 50–65 thousand years ago [4]. Thus began the interaction between humans and a largely endemic fauna that at first contact included megafauna [5].

**Figure 1:**
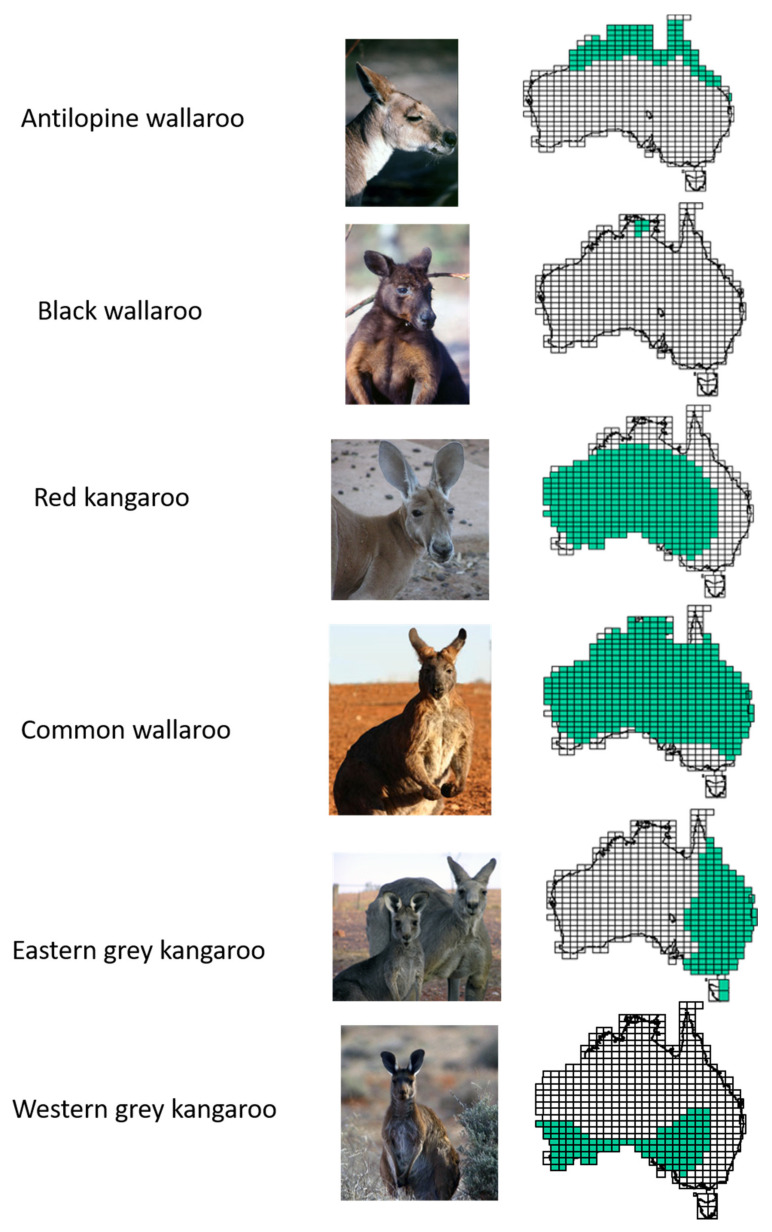
The six species of kangaroo and their distribution based on presence in 1:250,000 maps of Australia (see http://www.ga.gov.au; accessed on 21 April 2021). All images by DBC.

This review focusses on the six largest extant species of the Macropodidae, which are defined as the “kangaroos” by Dawson [6]. However, the term is loosely used to represent a conflation of several of these species or the whole family, leading to lack of precision in discussion of the issues—abundance, conflict, and conservation—canvassed in this review. Thus, an overarching aim is to address a habit of over-generalisation in the literature, especially its public face. These six species, red kangaroo (*Osphranter rufus*), common wallaroo (*O. robustus*), antilopine wallaroo (*O. antilopinus*), black wallaroo (*O. bernadus*), eastern grey kangaroo (*Macropus giganteus*) and western grey kangaroo (*M. fuliginosus*), collectively occupy most of the Australian continent and some larger offshore islands (Figure 1), and so the relationships of humans and the kangaroos are far from uniform across the continent. Collectively, the ranges of these kangaroo species span climatic zones as diverse as cool temperate, temperate, arid, and seasonally dry and wet tropics.

The abundance of some species in some parts of their range is measured with some precision and high frequency, especially in southern Australia (e.g., [7]), whereas other population surveys are infrequent and localised, such as in northern Australia (e.g., [8]). Species are variously described as abundant, over-abundant, super-abundant, and in “plague proportions”. There is also contention about historical changes in species’ abundance ([9,10]). Thus, the first aim of this review is to tease out the logic of the various perceptions of “abundant” kangaroo species and provide an historical perspective from first occupation by Aboriginal peoples to second occupation (also referred to as colonisation or invasion) by people from the British Isles and many subsequent nationalities from 1788. The second occupation resulted in significant displacement, some inter-breeding and eventual co-mingling with the first peoples.

The interpretation of conflict should have more clarity. If fauna provide ecosystem services, then those effecting disservices produce conflict even if this is entirely anthropocentric [11]. For the kangaroo species, the disservice is typically to diminish agricultural production, especially the profitability [12] and sustainability [13] of pastoralism in the Australian rangelands. Further conflict arises where a kangaroo species may diminish the prospects of one or more other species of higher conservation significance (e.g., in a threatened species category) [14]. These conflicts lead to the implementation of measures to control abundance. Typically, such a response is defined in the dimension of human–wildlife conflict. However, contention over the necessity and means of control also provokes significant human–human conflict [15,16]. Thus, the second aim of this review is to explore the nature of conflicts arising with the various kangaroo species at the interfaces between people and kangaroos. Furthermore, we explore the tensions between the various biological disciplines as to whether the individual or the population guides management (and the needs thereof) as exemplified in a compassionate approach [17,18,19].

The conservation status of the six kangaroo species is one of “Least Concern” [20]. Thus, conservation actions for the benefit of at least the four species with a southern and central Australian distribution are typically deemed unnecessary. The focus is on reducing their abundance rather than any incremental action. Their abundance and grazing are viewed as a threatening process in conservation management in protected and other areas, e.g., [21]. Thus, the final aim is to explore the generality of this contention and the interplay between studies showing benefits or benign effects of kangaroo populations, and those promoting a contrary result.

To fulfil these aims and give it currency, the review mainly explores peer-reviewed scientific literature from the last two decades in the disciplines of conservation biology, ecology and behavioural ecology. However, some topics require a broader reach back in time and into other disciplines such as history, social sciences, anthropology and archaeology. Further pertinent information is found in publicly available reports by or to regulators of kangaroo management programs. The public face of conflict and conservation with kangaroos is found in authoritative news articles such as those reported on the Australian Broadcasting Corporation News site (http://www.abc.net.au/news; accessed on 21 April 2021), the Guardian online news (http://www.theguardian.com/au; accessed on 21 April 2021) and the online platform for academic/researcher discourse, “The Conversation” (http://www.theconversation.com/au; accessed on 21 April 2021). There are numerous social media and website platforms that promote viewpoints about kangaroo conservation and management, but these are not referenced unless they host significant reports. We have canvassed many of the issues raised in this review in a 2005 publication directed at a general readership [10,22,23]. We apply academic rigor here and temper our own hyperbole.

## 2. Abundance

The extant kangaroo species have an evolutionary history that pre-dates the arrival of humans on the Australian continent by a million (*Osphranter* spp.) to two million years (*Macropus* spp.) [6]. Their populations have waxed and waned and their distributions expanded and retreated, no doubt many times, with dramatic climate changes and the dissolution of Sahul into Australia and New Guinea and the separation by sea-level rise of Tasmania from the Australian continent. Thus, the frequent claim that there are more “kangaroos” [24] than ever requires a reference point.

### 2.1. Aboriginal Australians

**Figure 2:**
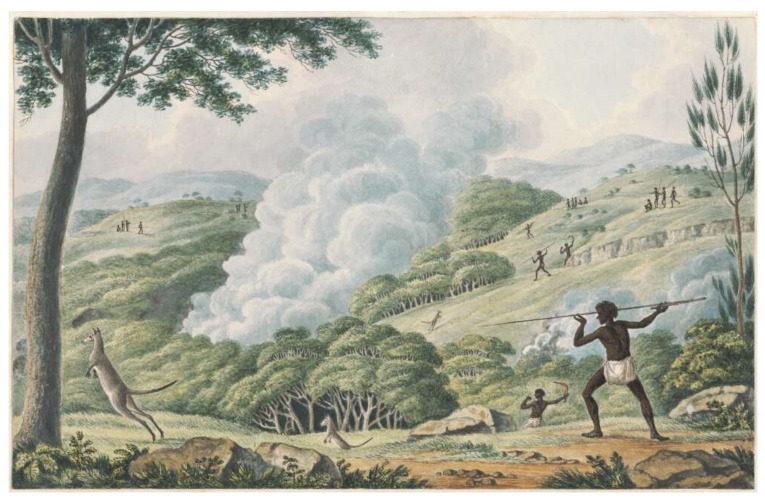
Aborigines using fire to hunt kangaroos by Joseph Lycett 1817 (source: http://nla.gov.au/nla.obj-138501179; accessed on 21 April 2021).

Gammage [25] describes Australian landscapes shaped by the precise and insightful use of fire by Australia’s first peoples. He summarises numerous accounts by European diarists and explorers, post-1788, that describe and, in some cases, paint (Figure 2) a park-like landscape. Signatures of such landscapes remain in the shape and form of very old trees (alive or moribund) in a few contemporary landscapes. Further studies have applied analysis of pollen, charcoal and dendrology to recreate a portrait of such landscapes which may have subsequently returned to forest in cool temperate Tasmania [26]. The use of fire to manage landscape ecology appears to be ubiquitous through Central Australia [27] to the tropical savannas of the top end of the continent. There, these practices, including securing water sources, can been gleaned from the knowledge of indigenous cultures and ongoing practices [28,29].

The deliberate introduction of fire into landscapes has been referred to as “fire-stick” farming [30], one of a suite of technologies that these first peoples used to manage resources and create an amenable landscape to transit on foot [31]. The practice could be used for immediate results, such as to cause prey to flee into the hunters’ ambit, or longer-term results, such as to regenerate plant food that attracted desirable prey. Many of these park-like landscapes supported the aptly named perennial kangaroo grass (*Themeda triandra*) which is very palatable to grazing herbivores (whether native or introduced) prior to seeding [25]. The post-fire attraction of various kangaroo species (and other macropodids) has been demonstrated in several studies in a variety of climate zones. For example, Denny [32] recorded a four-fold increase in the density of red kangaroos on Mitchell grassland (*Astrebla lappacea*) in an arid rangeland after fire, Southwell and Jarman [33] reported an immediate increase in the density of eastern grey kangaroos on a burnt mixed tussock grassland in eastern Australia in advance of coexisting cattle, and Gould [34] described the firing of spinifex in Western Australia prior to expected rain and the return to such areas when green shoots attracted kangaroo species. The effect may be conditional on rainfall distribution (e.g., western grey kangaroos in a mallee woodland [35]). The additional stimulus of moisture/rainfall is well-known in contemporary fire management in northern savannas [36]. In this environment, Murphy and Bowman [37] tested the hypothesis that fire and moisture attract kangaroo species (antilopine, black and common wallaroos) and a wallaby (agile wallaby *Notamacropus agilis*). Their proxy measure of the utilisation of burnt/unburnt habitats, macropodid faeces (scat), did not discriminate between species. Even so, they found a clear attraction to moist burnt habitats but away from burnt, dry, rocky ones. Bowman and his colleagues [38] have concluded from this and other studies in northern savannas that Aboriginal use of fire promoted hunting success, with various kangaroo species, by stimulating “green pick” (re-shooting grasses). They suggested “fire-stick” farming is in effect “fire-stick” ranching for the acquisition of game. Codding and colleagues [39] examined the relationship between fire management and the hunting of the common wallaroo by Aboriginal people in the western deserts of Western Australia. They found that “fire-stick” farming and an intermediate hunting pressure resulted in the highest population density of this species.

The centrality of kangaroo species (and other macropodids) in the diet, accoutrements, ceremony, art and mythology of Aborigines has been discussed by many authors. For example, diet includes a wide diversity of animal and plant matter, but kangaroos have probably always been an important and valued source of meat. There is a reward of many kilograms of meat for the hunting effort with the large species. Altman [40] found that the Gunwinggu of western Arnhem Land (wet/dry tropics of northern Australia) consume some 90 species of animals and 80 species of plants. Mammals provide up to 84% of energy intake in the mid-wet season and 91% in the late dry. Of the mammals consumed, seven of the fourteen species are macropodids. Kangaroos also provided the Aborigines with other valued resources in addition to meat. O’Connell [41] describes the manufacture of kangaroo-skin water bags in central Australia. Meagher and Ride [42] list the use of skins for cloaks and water bags, bones for personal decoration and awls, sinews of the tail for sewing and binding, and teeth for making scrapers. Recently, bone tools from a kangaroo species dating back 35,000 years have been uncovered [43]. In art, the oldest (17,000 years) Aboriginal painting on a rock face is of a kangaroo species [44].

Aboriginals demonstrate in behaviour and mythology an acute knowledge of the behaviour and ecology of the species they used (e.g., red kangaroo ecology in central Australia [45], ecology of rock-wallabies, black and common wallaroos in west Arnhem Land [46]). This wisdom is a necessity for a successful hunt. Liebenberg [47] has argued that the origin of the scientific method may reside in the principles of tracking followed in our hunter-gather past. From his observations, mainly of San people in the Kalahari, he determined that trackers classify and interpret their target’s spoor and formulate hypotheses that they test through further observation. Thus, they pursue a scientific method and Aboriginal peoples, known for their exceptional tracking abilities, no doubt do the same. Aboriginals also differentiate species and sexes and size classes of prey much as a behavioural ecologist would classify the population. For example, in the Gunwinggu language we find *Kandakeet* (male) and *Kandite* (female) for the antilopine wallaroo and *Kalperrt* (male) and *Wblerrk* (female) for the closely related common wallaroo [40].

Thus, in Aboriginal Australia, landscapes were managed to support the abundance of key animals such as the kangaroo species. This strategic and purposeful land management was likely ubiquitous and adapted to the locality (fauna, flora, climate, landform) [25]. It was not without error. There were extinctions in the Pleistocene megafauna. Killing by Aborigines was a likely cause [48,49], but this is disputed [50,51]. Competition between Aborigines and the largest indigenous carnivores for prey may have seen the latter extirpated from mainland Australia [52], earlier than the entry of potentially competing dingoes (*Canis dingo*) [53].

### 2.2. “European” (Post-Colonial) Australians

The colonisation of Australia by peoples with a European ancestry commenced with the arrival of the first fleet at Sydney cove on 26 January 1788 [54]. The “invasion” was a modest contingent of convicts and their administrators and guards. The British made a sovereign claim over the eastern half of the continent and the colony of New South Wales was proclaimed. The impact on the local Aboriginal population may have been immediate but on a continental scale it went well into the 19th century with successive waves of immigration. The initial relationship of this British colony with the kangaroo fauna was one of consumption by necessity. The initial novelty of kangaroos (eastern grey kangaroos at this location) waned, and the colonists turned their interest to the more mundane but pressing need of acquiring food. Thus surgeon Arthur Bowes Smyth commented in 1788: “There are great Nos. of Kangaroos but so extremely shy that ‘tis no easy matter to get near enough to them even to shoot them. As there is a most exact print of this uncommon Animal in Capt. Cook’s Acct. of this Country I shall not take the trouble to describe it” (p. 58) [55]. The culinary value of kangaroos was not highly rated. Watkin Tench writing in 1778 remarks: “Of the flesh we always eat with avidity; but in Europe it would not be reckoned a delicacy: a rank flavour forms the principal objection to it… The tail is accounted the most delicious part, when stewed” (p. 268) [56].

When the colony became established and more reliant on domestic stock, kangaroos were hunted more for recreational than culinary reasons. A special breed of “kangaroo dog” was selected from the greyhound and a more powerful breed such as the mastiff. Of the sport, Oxley in 1820 [57] enthuses: “I think that most fastidious sportsmen would have derived ample amusement during our day’s journey. He might have seen the truest coursing from the commencement of the chase to the death of his game without moving, and tiring of killing kangaroos he might have hunted emus with equal success.”

From the establishment of livestock production in Australia and its spread across the continent, the popular narrative is that kangaroo species have never had it so good. They are abundant, overabundant, super-abundant, in “plague proportions”. The concept of unnaturally abundant kangaroo species became entrenched with Newsome’s 1975 article [58] on “their [red kangaroo, common wallaroo] anomalous prosperity since the introduction of ruminant stock to their environment”. The anomaly is that these species along with the eastern and western grey kangaroos have persisted despite often brutal and sustained killing. The post-colonial mammal fauna of Australia has suffered mass extinction [59], including eight species from the Macropodidae and related Potoroidae. Many more have suffered significant contractions in their historical ranges [60]. The exception to this trend is not a kangaroo but the swamp wallaby (*Wallabia bicolor*) which persists in peri-urban woodlands around major east coast cities [61] and has expanded its range in many rural areas [62].

The four main factors cited for unnaturally abundant kangaroo species are: (1) displacement of Aboriginal populations and their hunting, (2) suppression of the dingo and its hunting, (3) clearing of forests and woodland to expand grasslands for grazing livestock, and (4) provisioning of water by impoundment and bore-sinking for livestock and the attendant human population. Johnson [63] summarises these contentions with a view from the 1860s frontier in relation to dingo regulation of an unnamed kangaroo species. For the initial increase in abundance, reliance is made on explorers’ accounts and diarists. Several authors read these accounts in support of the assumption [64,65,66]; others disagree [67,68]. Our contention is that it is one of many over-generalisations about kangaroo species and it ignores the fate of northern Australian species, which have declined in abundance as discussed below.

If the first peoples managed the landscape for kangaroos with the clearing and maintenance of grasslands, then this should have had an increaser effect. This may have been counter-balanced by Aboriginal hunting success and the predation by, first, marsupial predators (thylacines *Thylacinus cynocephalus*, Tasmanian devils *Sarcophylis harrisii*), and then dingoes. This would fit Johnson’s thesis, but were Aborigines accommodating to competing predators? Prowse and colleagues [52] modelled the interactions between predators (humans, thylacines, Tasmanian devils, dingoes) and prey (macropodids) under climate change in late Holocene Australia. They concluded “human intensification” (a combination of population increase and technological advance), under a more variable climate, likely caused mainland extinction of thylacines and devils. Likewise, Fillios and colleagues postulate that the dingo was both a facultative companion and competitor [69]. Furthermore, the motives and attention of explorers and diarists were likely quite variable in relation to the indigenous fauna. One would expect the remarkable to take precedence to the mundane.

One explorer, John Oxley [57] sought landscapes suitable for pastoral production and judged these on the abundance of “game”. In his explorations of the central west of New South Wales, he provides numerous accounts of abundant kangaroo species (most likely eastern grey kangaroos) and the macropodid and potoroid diversity at the time. In his very first mention on 25 May 1817 near current-day Goulburn, Oxley writes: “We have seen so few animals, either kangaroo or emu, and the country seems so little capable of maintaining these animals, that the means of the natives procuring food must be precarious indeed” (p. 46). This statement is married with one about the poor condition of the country, and on May 31 he writes: “No herbage of any kind grew on this abandoned plain, being a fine red sand, which almost blinded us with dust” (p. 50). From these writings, you could argue that there were few kangaroos but, under the same conditions today (no herbage), there would likewise be few, if any kangaroos. Oxley was in fact an excellent observer of what we would now call the ecology of this system. Thus, in contrast, he later writes on 6 August 1818: “We killed this day the largest kangaroo we had seen in any part of New South Wales; being from one hundred and fifty to one hundred and eighty pounds weight. The animals live in flocks like sheep; and I do not exaggerate, when I say that some hundreds were seen in the vicinity of this hill; it was consequently named Kangaroo Hill: several beautiful little rills have their source in it but are soon lost in the immeasurable morass at its base” (p. 258). Oxley is just to the west of the current day Warrumbungles National Park which is noted for its large population of eastern grey kangaroos. This observation precedes any livestock, water management, dingo management or displacement of, in Oxley’s term, natives. In relation to dingoes, he notes on 7 June 1817: “These ranges [near present day Goulburn] abound with native dogs; their howlings are incessant, day as well as night…” (p. 59).

Hunting likely had a lesser impact on kangaroo populations than the killing that followed the identification of kangaroo species as pests to the pastoral industry. Once pastoral enterprises grazing sheep and cattle were established, the conflict began. In 1889, Neville-Rolfe [70] writes: “A plain, stripped of all grass by the invading hordes, brown, too, with the figures of four or five hundred of the enemy, who, on first appearance of a human being, dispersed in all directions, and with rapid bounds passed away into mere specks on the rolling downs. The poor starved sheep, wondering where all the grass had gone, and why it did not come after the rain as of yore, were unable to copy the hardier kangaroo, who, if he never grows fat, can live on country where the domesticated animal must die” (from [71], p. 312).

By the 1880s, all eastern Australian states had enacted legislation that encouraged the eradication of kangaroos. Kangaroos and wallabies were declared vermin in NSW in 1880. Neville-Rolfe (from [71], p. 312) states that “a price varying from one shilling to threepence was put on the head of each grass-eating marsupial, of which the Government pays one half and the district in which the marsupial is killed the other half”. Denny [32] reviews several sequences of red kangaroos killed in very large numbers prior to contemporary population survey methods. For example, in the decade 1880–1890, which was also coincident with both drought and the payment of bounties, the estimated population in New South Wales reduced by 4.5 million (5,484,000 to 921,500). At that time, concern was raised by some commentators as to the sustainability of this control. For example, Saville-Kent [72] states: “Being essentially vegetarian in their habits, the larger species of the kangaroo family, where abundant, so seriously tax the resources of the Australian pasture lands as to necessitate the adoption of stringent measures to keep them in check. This untoward necessity, combined with the high value set upon kangaroo skins, has contributed towards the complete extirpation of the ‘Boomer’ throughout a large extent of the prairie-like tracts of Australian pastoral land on which it abounded previous to the advent of the settler” (p. 21).

By the 1950s, concern was again raised by pastoralists about large numbers (“plague proportions”) and the population was reduced by 75% by the 1960s. Saville-Kent’s concerns were again echoed in the 1960s, when severe drought caused high kangaroo mortality and kangaroo harvesting was seemingly out of control as unemployed station (ranch) hands sought alternative income. The conservation voice was heard stridently for the first time. Montgomery [73] wrote “Is it too late to save the Big Red?” in a popular international journal, *Animals*. He lamented that “Australia’s national emblem is in danger”. Readers were confronted with photographs of massed kangaroo carcasses. This triggered intervention by the government in the form of protection and the regulation of an industry killing kangaroos for meat and skin [74,75]. This industry persists today, with meat used for both human consumption and pet food, unlike the founding industry, which was pet-food based. Commercial consumption replaced subsistence in the late 19th and early 20th centuries with a skin trade associated with the period of payment of bounties. Kirkpatrick [74] writes “The enormous supply made available by the pest destruction operation was exploited by shooters and dealers who extended this into an industry even in Western Australia before the agriculturalists and pastoralists developed there” (p. 79). His last statement is suggestive of plentiful kangaroos in Western Australia prior to livestock.

### 2.3. Present-Day Australia

We are again in a period where various kangaroo species are considered in excessive numbers. Talk of “plague proportions” is less frequent and has been replaced by “overabundant” kangaroos regardless of species or location. The first comprehensive book on kangaroo ecology and management by Frith and Calaby in 1969 [64] already considered “plague proportions” as propaganda not fact. They write “…some landholders’ organisations have coined a useful phrase ‘plague proportions’ to cover most kangaroo populations and refer to greater local numbers than existed locally last year, last decade or, in some cases, last century, to justify further reduction in numbers” (p. 60).

During extended dry periods, isolated rainfall events can attract red kangaroos from a large catchment area (~50 km) and concentrate them at extremely high local densities. The duration of such a mass influx is a month or less. Denny [32] recorded events on Sturt National Park in far western New South Wales with a 270-fold increase in abundance, Edwards et al. [76] captured an event on the University of New South Wales Arid Zone Research Station at Fowlers Gap about 220 km south, and IW recorded an event where red kangaroo density in a 991-ha study area increased from 20 to over 100 km^−2^ for a month at Fowlers Gap [23]. This behaviour is specific to red kangaroos. The density of the other common species, western grey kangaroo, remained unchanged on IW’s study site.

Herbert [77], in a review of contraceptive control of reproduction in eastern grey kangaroos, notes “…it is very difficult to define the term ‘overabundance’. It is largely defined by human interests and as such it tends to involve subjective, value-laden judgements…” (p. 67). Overabundance (or overpopulation) implies obviously too many individuals of a species, which are either causing harm to themselves (e.g., injurious fighting, starvation), harm to their ecosystem (e.g., landscape dysfunction, endangerment of other species), harm to people (e.g., vehicle collision, monetary loss, threats to livelihood) or any combination of these [78]. A typical kangaroo example is a small peri-urban reserve. The species will almost inevitably be an eastern grey kangaroo, the population will grow, forage will be depleted, some individuals will starve and die as they cannot easily disperse, others will eat the neighbour’s lawn or collide with their car. The reserve will most likely be on old farmland with “improved” pasture invaded by weeds. The exotic grasses will no longer be supported by copious application of phosphate fertiliser so the system is inherently unstable, and collapse should come as no surprise. This scenario is played out in Australia’s capital, Canberra [79,80,81].

The focus of the postulate of over-abundance is typically that a single species or collective (up to four species may overlap in distribution in far-western New South Wales) does harm to itself, harm to the ecosystem and harm to the economy [82]. The latter refers to the complexity imposed on livestock managers by a variable “unmanaged” population of one or more kangaroo species. This may manifest in the inability to meet “total grazing pressure” [83] targets at the paddock and/or property level. In simple terms, total grazing pressure is defined in the above citation as “the total forage demand of all vertebrate herbivores relative to the forage supply”. It is a simple accounting of the offtake of each herbivore without reference to synergies and successional grazing as expected in a natural herbivore guild. There is no reference to other herbivory (e.g., grass-eating termites that are diverse and abundant in Australia [84]), except by the assumption that this is discounted at the time of measurement. The interactions between the herbivores are complex because both the indigenous marsupial fauna (losses to local or total extinction [10]) and the flora of the forage (introduced grasses, herbs and weed species) have dramatically changed. Dependent on one’s location in Australia, in extensive grazing systems one or more species of kangaroo may graze with wallabies (including rock-wallabies), rat-kangaroos (Potoroids), wombats, sheep, goats, cattle, swamp buffalo, Bali banteng, one-humped camels, horses, donkeys, deer (one or more of six species), South American camelids, rabbits, brown hares, as well some omnivores such as native bandicoots and introduced pigs. The grass layer may include species from any continent except Antarctica. Such has been the pervasiveness of this disruption of Aboriginal Australia that few if any pristine areas exist. In this mix, the large, abundant and obvious kangaroo species are sometimes embraced but more often demonised as causal to or scapegoated for the mismanagement of collapsing ecosystems [85] or the failure to restore previously degraded landscapes in protected reserves or grazing land in the rangelands [86].

In Australia, the stocking rate for a pastoral property is estimated as dry sheep equivalents (DSE), i.e., a non-lactating ewe of average size. This provides a common metric to assess the potential grazing pressure of livestock and two species of kangaroo (Table 1).

**Figure 3:**
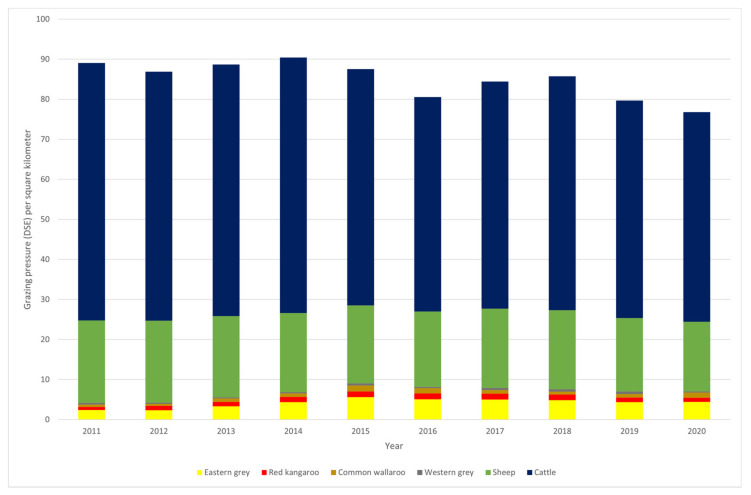
An estimate of annual grazing pressure by cattle, sheep, eastern grey kangaroos, red kangaroos, common wallaroos and western grey kangaroos based on stocking rate equivalents (DSE) per km^2^.

The abundances of four species of kangaroo are estimated in the kangaroo management zones of five states (Queensland, New South Wales, Victoria, South Australia and Western Australia). The Victorian estimates do not cover the last decade and so are not included in the following analysis. The kangaroo abundances are estimated over a static area (excluding increases in management zones in South Australia and New South Wales in the latter part of the decade) where the species is counted. These areas are: red kangaroo—3,863,816 km^2^ (all four states), common wallaroo—1,729,419 km^2^ (Queensland, South Australia and northern tablelands in New South Wales), eastern grey kangaroo—1,685,520 km^2^ (Queensland and New South Wales), and western grey kangaroos—2,733,205 km^2^ (Western Australia, South Australia, western plains in New South Wales). The abundances of the four kangaroo species are reported at https://www.environment.gov.au/system/files/pages/ee20f301-6c6c-44e4-aa24-62a32d412de5/files/kangaroo-statistics-states-2020.pdf (accessed 19 April 2021). For cattle and sheep, annual statistics are provided in the agricultural statistics of the Australian Bureau of Statistics (https://www.abs.gov.au/statistics/industry/agriculture; accessed on 19 April 2021). In most of these surveys, the land area devoted to predominantly grazing is estimated and ranges from 2,694,570–2,813,220 km^2^ in the period 2011–2020. Thus the densities of four kangaroo species, sheep and cattle were estimated and multiplied by a simplified DSE (kangaroo 0.35, sheep 1, cattle 8) to examine trends in grazing pressure across the last decade (2011–2020) (Figure 3). Clearly the kangaroo species, individually or collectively, exert a minor proportion of the grazing pressure relative to the two livestock species grazed within their ranges.

## 3. Conflict—Four Kangaroo Conundrums

The human–wildlife and human–human conflicts relating to kangaroo species are referenced to the four purported causes of their “unnatural” increase: human predation decline, dingo predation decline, pasture/forage improvement and watering point expansion and stability (e.g., [89]).

### 3.1. Human Predation Decline

Williams [90] estimates an Aboriginal population of 0.77–1.1 million at the time of European contact. Subsequently, Bradshaw and colleagues [91] have estimated a population of 3.1 million. The average “kangaroo” carcass taken by the commercial industry yields 23 kg of meat [92], but realistically Aboriginal hunters were more likely to kill the “average kangaroo” with a yield of 13 kg of meat. The recommended diet of red meat is 250 g weekly. At the lower limit, 0.77 million Aborigines eating large kangaroos (23 kg meat) would kill 435,217 per year. At the upper limit, 3.1 million Aborigines eating average sized kangaroos (13 kg meat) would kill 3.1 million per year. Modern accounting is here applied to historical consumption. There are both over-estimates (the Aboriginal “larder” included many more species) and under-estimates (spoilage) in this simple calculation. It is also notable that a moderate hunting effort in combination with landscape management (“fire-stick” farming) may enhance not suppress a kangaroo species’ population [93]. The current commercial killing of kangaroo species operates in five states—Queensland, New South Wales, Victoria, South Australia and Western Australia—and allows an annual quota that exceeds 3.1 million kangaroos. However, only four species are included (red kangaroos, eastern and western grey kangaroos, wallaroos) and the combined quota of these independent management programmes is not reached. For example, in the most recent year (2019) for which complete values (excluding Victoria) are available, the summed quota is 6,436,686 and the kill is 1,570,473 (https://www.environment.gov.au/system/files/pages/ee20f301-6c6c-44e4-aa24-62a32d412de5/files/kangaroo-statistics-states-2020.pdf; accessed on 19 April 2021).

This suggests that current human predation, at the upper limit of our calculation, is in arrears of Aboriginal Australia but it does not tell the full picture. Firstly, the commercial zones cover a minor proportion of the continental area. The hunting pressure includes non-commercial killing in the Australian Capital Territory, “destruction” permits in all jurisdictions, legal and illegal recreational hunting, and unrestricted Aboriginal (indigenous) hunting that add to the kill figure from the commercial industry but cannot be fully accounted for. In addition, roadkill of kangaroo species is substantial (e.g., [94]) across all jurisdictions and there are other anthropogenic causes of mortality such as entanglement in livestock fencing. Thus, all the anthropogenic sources of kangaroo mortality are currently likely to exceed the former killing by Aborigines, which itself has not completely ceased. To meet this end may justify the operation of a lethal reduction in the abundances of the various kangaroo species but it does not explain the purported excess in their contemporary abundances, even if the commercial industry is not meeting some stakeholders’ perspectives [82]. There are of course additional causes of mortality such as starvation induced by drought, competition or both [95], and disease [96]. There may also be density-dependent suppression of breeding success [97,98]. There is also non-human predation (e.g., [99]). Where an annual census of the abundance of species is conducted, then these sources of mortality (anthropogenic and non-anthropogenic), constraints on breeding, and immigration and emigration into the survey areas are accounted for.

### 3.2. Dingo Predation Decline

**Figure 4:**
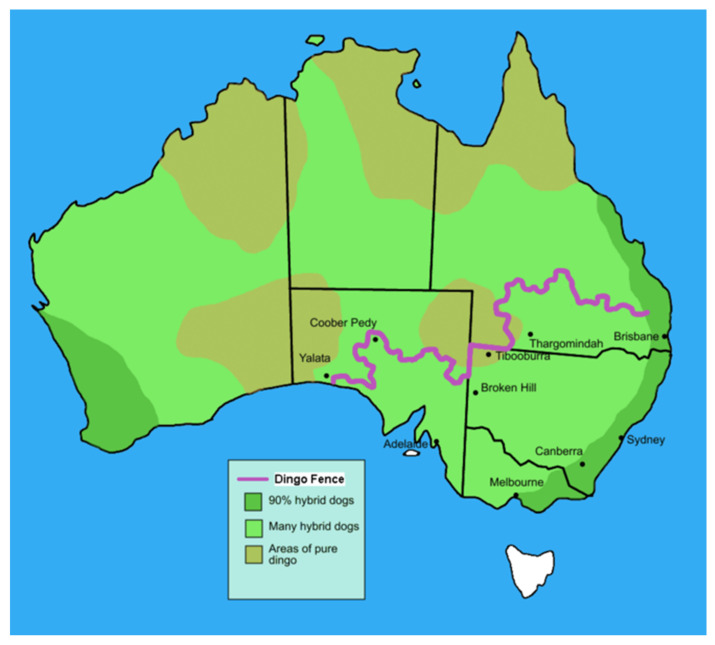
Dingo (or wild dog) barrier fence across Queensland, New South Wales and South Australia (source: https://commons.wikimedia.org/wiki/File:Dingo_fence_in_Australia.PNG; accessed on 21 April 2021). Note, there is a non-contiguous barrier fence (not shown) from the south-east to the north-west of Western Australia.

The interpretation of the ecological role of the dingo, particularly the regulatory effect of its predation on kangaroos, is based on studies of areas with or without significant residual populations of this predator. On a large scale this is based on interpretation of the abundance of kangaroo species on either side of one 5614 km barrier fence (Figure 4) (there is a similar fence within Western Australia). This eastern-central fence was erected in the late 19th century to prevent the northwards progression of rabbits (to which it proved ineffective) and then found utility (with some raising of height) as a control on dingoes (wild dogs), which is its current function. Following construction, there was a concerted effort to kill dingoes on the southern (inner) side and large-scale baiting/trapping programs continue in many jurisdictions today. Johnson [63] provides a comprehensive review to the time of the publication (2015) and concludes that comparative studies of dingo and prey populations support the suppression of kangaroo species’ populations where dingoes are abundant. Furthermore, the role of the dingo as an apex predator and trophic regulator has been promoted in several studies (e.g., [100]), but not without debate [101].

Caughley [102] contended that dingoes controlled the populations of kangaroos through aerial surveys across the fence in the vicinity of Tibooburra (see Figure 4). Newsome [103] initially supported that conclusion but retracted it after further examination of the vegetation across the dingo fence [104]. Newsome’s title about two universes on either side of the fence is compelling given a divergence in grazing history of more than a century with sheep south of the fence and cattle to the north, and summer rainfall to the north and predominantly winter rainfall to the south. Denny [32] demonstrated a strong fence-line effect in the corner between New South Wales and Queensland, and New South Wales and South Australia. He noted a tendency of red kangaroos to graze into the wind, resulting in at least a two-fold increase in density on these boundaries. Added to this are potential mass movements to the northwest in response to rainfall on the outer (north/west) side of the fence, leading to transient but very high densities against the fence. Johnson [63] contends that these small-scale differences have been resolved by more recent large-scale studies. The most recent of these uses remote sensing [105] and concludes that there is a pronounced depletion of vegetation cover and quality within the fence (south and east) where dingoes are suppressed compared to outside (north and west) for a common arid dune-swale system. They use Morris and Letnic [106] to draw a causal relationship with grazing by red and eastern and western grey kangaroos based on the exclusion of these species from plots and a bold assumption that grazing by rabbits was equivalent inside and outside plots. The kangaroo grazing is predominantly driven by red kangaroos as eastern grey kangaroos are not found outside and further west of the fence, and in our collective long experience in Sturt National Park are unlikely in the west on the Strzlecki dune fields. The generality of the remote sensing study is questionable because the study area of Sturt National Park is very complex, with intense and unresolved degradation from livestock grazing [107]. The Park itself is “two universes”—the dune fields in the west with largely ephemeral grazing and the stony downs in the east with perennial Mitchell grasses.

There is no doubt that dingoes prey on kangaroo species along with a range of other prey [108]. They may engage in overkill as recorded in Sturt National Park [109]. Dingoes are not ubiquitous in contemporary Australia and their identity among hybrids with domestic dogs and feral dogs adds complexity [110]. They are more common in northern Australia, but other factors seemed to be at play in suppressing kangaroo species there, as discussed below.

### 3.3. Pasture/Forage Improvement

**Figure 5:**
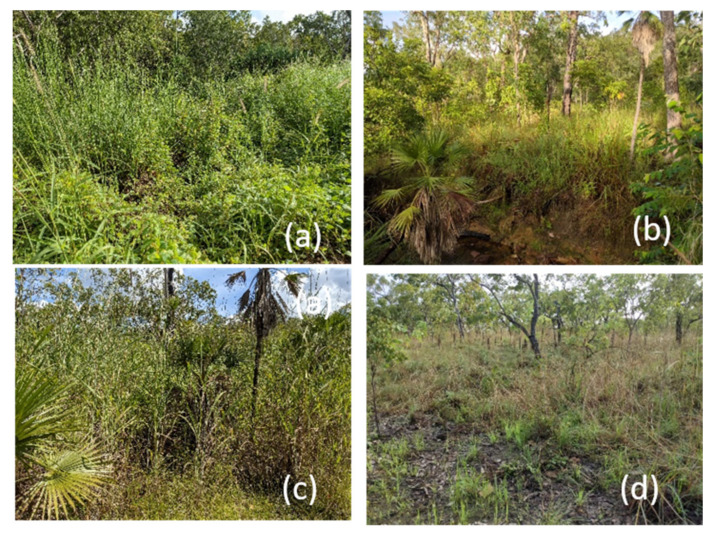
Landscape grazed by antilopine and common wallaroos infested by unpalatable species from a prior pastoral use. (**a**) Invasive annual (*Cenchrus pedicellatus*) and perennial (*Cenchrus polystrachios*) mission grasses (and other herbaceous weeds) in a riparian zone, (**b**) Riparian grassland of native species, (**c**) Invasive gamba grass (*Andropogon gayanus*) amongst native sand palms, and (**d**) Grassy understory of native savanna woodland. (All images by DBC).

It is contended that land clearing expanded grasslands, and pastures were improved (less grazing sensitive species and/or application of fertilisers), leading to an overabundant kangaroo population of all or any of the six species. This hypothesis suits a narrative that European farming practices and the introduction of livestock enabled the populations of kangaroo species to prosper and thus their suppression to a lower density is within the provenance of the landholder. The evidence is not compelling. As discussed above, Aboriginal Australia was managed to the kangaroo species’ benefit. In contemporary Australia, the kangaroo species have been excluded from parts of their former range by urban development and expansion, leaving vulnerable populations [111,112], significant tracts of land have been taken for horticulture and cropping, leaving isolated populations [113] at significantly lower densities than the rangelands [114], there has been significant degradation of the southern rangelands, leading to adverse shrub incursions and loss of drought resilience from perennial vegetation and reliance on an ephemeral bounty [115], and there has been the replacement of highly palatable annual and perennial grasses with unpalatable (to kangaroo species) grasses from Africa and elsewhere, leading to weed invasion [93,116]. An example of the latter is gamba and mission grasses (Figure 5) in northern Australia [117,118] that are unpalatable to native herbivores, transform landscapes through intense hot fires relative to native grasses, and displace more palatable and beneficial native grasses. They have been declared “weeds of national significance” and threatening processes to biodiversity. Their control has become so intractable that Bowan [119] provocatively suggested the introduction of elephants to Australia to suppress them.

Sheep grazing has been identified as a threat to native biodiversity and explains many past declines in marsupial species [120]. Reciprocal competition between sheep and red or grey kangaroos was predicted by Caughley and colleagues [121] and demonstrated by Edwards and colleagues [95,122] for red kangaroos. Such competition was intermittent and intensified with rainfall deficits and sparse pasture conditions. At such times, sheep foraging with red kangaroos had lower live weights but no deficit in wool production. Red kangaroos segregated from sheep into destocked paddocks but did not show poorer condition in their presence. IW [123] later showed a pronounced segregation of red kangaroos from sheep in the same site during lambing when the flock is dispersed across the paddock. A radio-tracking study of red kangaroos by Norbury and Norbury [86] in the Western Australian rangelands likewise found local individuals moving into an area where sheep were removed. They further found that such behaviour diminished the regeneration of forage in a term of 4 years [124]. This was not the case in the prior two studies where significant regeneration of the shrub layer had occurred over much longer destocking (up to three decades). Tyndale-Biscoe [125] reviewed the relationship between sheep and kangaroos and provocatively called it “an unequal contest” with kangaroos the loser.

Newsome [126,127] demonstrated a tendency for red kangaroos to segregate from cattle in central Australia. He later predicted [58] that cattle grazing would be more detrimental to kangaroo species than sheep and the combination of the two of higher impact. In a study in central Australia, the removal of cattle grazing led to an increase in red kangaroo numbers, but landscape effects were confounded by the presence of camels [128]. In the northwest of Australia [129,130], cattle grazing significantly reduces populations of antilopine wallaroos. Ritchie and colleagues [131] demonstrated a significant negative interaction between eastern grey kangaroo abundance and cattle abundance in north-eastern savanna.

The causation of a presumed competitive interaction between one or more kangaroo species and livestock can be misunderstood. In the Pilbara region of northern Western Australia in the 1960s, “war” was declared on the euro (common wallaroo) population, which burgeoned while sheep production and red kangaroo populations declined [132,133,134]. Competition was assumed between the common wallaroo, its congener and sheep that could be resolved by mass killing of the common wallaroos. The research by Ealey and his colleagues revealed that sheep grazing had degraded palatable species from the pasture to the disadvantage of both sheep and red kangaroos. The common wallaroo, with its capacity to maintain itself on poor quality pasture (dominated by spinifex) through adaptations such as nitrogen recycling through the saliva [6], sustained its population.

### 3.4. Watering Point Expansion and Stability

**Figure 6:**
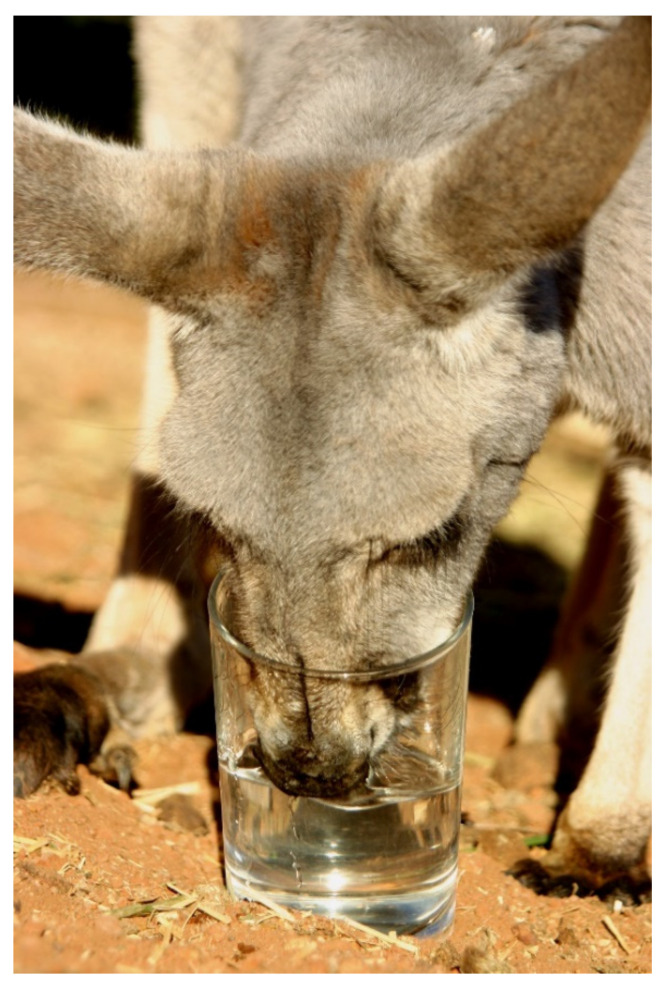
Red kangaroo drinking from a small narrow water source which would be inaccessible to a like-sized sheep or cow. (Image by Ulrike Kloecker reproduced with permission).

In the rangelands, water has been secured in off-stream storages through diversion into earthen impoundments (referred to as tanks) or subterranean sources have been tapped by bore sinking. These sources (referred to as artificial watering points) secure water supply (although the tanks can evaporate dry in drought) and may supply a large perimeter to drink from. This is essential for livestock and human occupants. However, the requirements and capabilities of the various kangaroo species are quite different. They have a long narrow muzzle and equally long tongue with which they lap water, making small ephemeral sources accessible (Figure 6). In contrast, sheep and cattle have broad muzzles and suck water and thus require relatively deep and broad water sources. If surface water is not available, common wallaroos will dig soaks in creeks, gaining access to further water that is not available to livestock. Furthermore, all the species of kangaroos need much less water than livestock. Graziers normally budget 10 L per day for sheep, with this potentially doubling in a hot dry summer. The water turnover in summer is 97 mL kg^−0.71^d^−1^ for red kangaroos, 104 for euros and 177 for eastern grey kangaroos [135]. This translates to around 1 L for a 25 kg red kangaroo or euro and 1.75 L for a 25 kg eastern grey kangaroo. Not all of this is required from drinking as water taken in with plant matter and created by the oxidation of foodstuff both add to the water budget. Wilson and Edwards [136] provide an estimate for daily water use that includes beef cattle (80 L), a generic kangaroo (1.5 L), and sheep (11 L).

Thus comparative physiology affirms kangaroo species are relatively miserly drinkers compared to livestock, and certainly people. They can exploit water sources that are small and shallow that are insufficient for livestock. DBC’s observations made during a study of drinking behaviour [137] revealed that they will readily lap muddy and algae infested water that is unacceptable to sheep. For kangaroo species, light rainfalls across the hotter months will maintain adequate ephemeral water sources in the many catchments from clay-pans and depressions (gilgais) to swamps and lakes. Many of these have been silted up by the much accelerated erosion introduced by livestock and overstocking [138,139]. Large water holes have been replaced, not necessarily supplemented, by earthen tanks. Many of these lie on top of old springs or simply create deep holes in existing swamps [140]. Not surprisingly, the distribution of kangaroo species inhabiting arid and semi-arid rangelands does not correlate with artificial watering points [141], red kangaroos do not show water-focused grazing [140], and various studies that have experimentally closed artificial watering points [142] show no significant effect on the distribution and abundance of the kangaroo species of interest, although the latter authors contend that the scale of these studies may be insufficient. Ritchie and colleagues [8] did find a positive relationship between antilopine wallaroo abundance and permanent water in the seasonally arid tropics of north Queensland but no relationship with eastern grey kangaroos and common wallaroos across their study area. Harris and colleagues [143] at a landscape scale (5 or 50 km resolution) found eastern grey kangaroos preferred mesic environments which likely historically contained permanent water prior to any livestock grazing. Red kangaroo and western grey kangaroo localities were related to permanent water but at long distances, suggesting travel and intermittent use. Thus, any causal relationship between the abundance of the various kangaroo species and the distribution of artificial watering points is at best weak.

## 4. Conservation—Six Species of Least Concern

Given the “least concern” conservation status of the species of kangaroo and their abundance, the focus in conservation biology research has been about costs and benefits to biodiversity. There is the general principle that grazing herbivores at an optimal density maintain an open pasture with a diversity of herbs and grasses [144]. In the absence of grazing, a few herbaceous species (fast-growing and palatable) will overwhelm the pasture and reduce diversity. If the grazing is too intense then pastures may be overwhelmed by unpalatable and often woody species. Grazing herbivores may also provide a service in fuel reduction in grassland fires [145]. Again, in a functional ecosystem, the herbivore guild creates a balance. Grazers reduce the grass fuel load, resulting in less fire damage to shrubs and trees. Browsers reduce the overshadowing foliage of shrubs and trees and thus maintain a more open habitat favouring grass growth. In Northern Australia, the introduction of tall African grasses (see Figure 5 above) without a guild of herbivores to control them has resulted in ecosystem dysfunction [118].

Sheep and, more particularly, cattle may occupy a vacant niche left by the extinction of the marsupial megafauna [48]. If this is true then sheep, cattle and kangaroos can live together as they occupy separate niches. If the megafauna extinction was a result of climate change and loss of habitat then there may be no vacant niches. Either way, based on modest body size, dentition and gut size, kangaroos should be at the more specialised end of the grazing gradient than larger sheep and cattle [146]. However, in the unpredictable environment of Australia, especially the rangelands, specialisation is risky. The rangeland kangaroos do show a preference for grass and winter forbs [6], but variability between individuals and environmental conditions in dietary niche is quite high [147,148]. They are not exclusive grazers, and the diet of western grey kangaroos can have a high browse component [149]. When you watch individuals eating, they sample a variety of plant types and their bipedal stance and long forearms allow them to reach up to pull down foliage. Likewise, the manipulative forepaws of all species allow access to shrouded grasses and herbs unavailable to livestock.

In addition to contributing to grazing and grazing diversity, the various species of kangaroos create diggings (hip-holes) that may beneficially modify the chemical and physical properties of soils through the entrapment of faeces and litter, and soil turnover [150]. At a high density (70 kangaroos km^−2^) in a peri-urban mesic environment, the grazing of eastern grey kangaroos was not detrimental to soil health [151]. In contrast, at a relatively higher density (not estimated), the grazing by red kangaroos and eastern grey kangaroos significantly reduced the soil nutrient (total carbon and nitrogen, available phosphorus) pool relative to grazing exclusions at a site with low kangaroo species’ density [106]. These authors argued this would mute vegetation responses to rainfall. On a flood plain, grazing eastern grey kangaroos contributed to local retention of energy (in the carbon pool) and the formation of energy sinks from faecal deposition within feeding sites, which was considered beneficial [152]

Although not studied in kangaroo species, other macropodids may assist the propagation of seeds through distribution off fur and in faeces (e.g., [153,154]). In contrast, grazing exclusion over three years in the dune fields of Sturt National Park increased grass seed production by 87% relative to nearby open plots [155]. The authors concluded a suite of one or more of four kangaroo species (individual species were not differentiated) grazing their study sites may reduce the abundance of small granivores, although none of these were sampled nor did they consider the propagation of grasses by any kangaroo species as a counterbalance.

Two recent studies [14,156] conclude that one or more kangaroo species are a threat to biodiversity in protected areas. The first conflates the grazing pressure by three species, red kangaroos and eastern and western grey kangaroos, and derives a generic index of kangaroo density in four conservation reserves which ranged from 145 kangaroos km^−2^ at Oolambeyan, 50 kangaroos km^−2^ at Yathong, 17 kangaroos km^−2^ at Mungo, and 7 kangaroos km^−2^ at Boolcoomatta. The reserve with the lowest density effected lethal control on their kangaroo species’ populations. All reserves had rabbits and feral goats and a past long history of livestock grazing, which was not considered by the authors, but at least in some instances led to the abandonment of unviable leases and re-acquisition for nature and/or cultural (Mungo) conservation. The areas were drought-affected and the authors concluded the role of reserves as a haven for kangaroos should be re-assessed and populations should be managed down (presumably by lethal means). The second study was conducted in the agricultural zone of South Australia. The estimate of grazing was as follows: “The extent and impact of grazing within the plot [30 × 30 m²] was measured over a 30 min period but no attempt was made to discriminate between the grazing impacts caused by different herbivore species” (p. 3). The authors’ main conclusion was that grazing pressure was of similar intensity in stocked and un-stocked (protected) areas, and that red kangaroos or western grey kangaroos or both were causal in the un-stocked reserves without measuring their density. The public presentation of this research was entitled “Kangaroos (and other herbivores) are eating away at national parks across Australia” from a study in one region of one state. The article includes statements that kangaroos (of no identified species) are the major contributor of impacts on some (not all) protected areas. These two studies typify the tendency to refer to a generic “kangaroo” whose combined effect is generalised across areas well beyond the bounds of the study. A further article provides commentary on this issue [157]. The crux seems to be that we should not expect conservation reserves to be a haven for any kangaroo species simply because they are native, and killed and extirpated beyond a reserve’s boundaries.

There are other small-scale studies that have employed lethal control of one or more kangaroo species for a conservation benefit. For example, the abundance of western grey kangaroos was reduced to assist avifauna in a RAMSAR site in Western Australia [158]. In 2019 in South Australia, western grey kangaroos and common wallaroos were killed in one or more of nine conservation reserves in ongoing programs for ecological restoration/threatened species recovery [159]. Eastern grey kangaroos are regularly killed in the Australian Capital Territory to support grassy ecosystem conservation [160]. In various contexts (e.g., urban development), non-lethal methods have been applied to reduce abundance; for example, translocation of eastern grey [161] and western grey [162] kangaroos, or fertility control of eastern grey kangaroos [163]. Ironically, livestock grazing may be recommended in some conservation reserves to manage invasive pasture grasses [164].

This debate about biodiversity loss (and potential recovery) often hinges on listing a series of threatening processes (or pressures) without due regard for proximate and ultimate causes, and the interactions between processes. A typical list includes land-clearing, introduced meso-predators (cats and red foxes), grazing by livestock and/or overabundant native herbivores (usually contemporary but sometimes historic), weeds, and changed fire regimes (too little or too much). Land-clearing is typically the ultimate factor and has accelerated in northern Australia, but a focus on killing animal “pests” or eradicating weeds conveniently draws attention away from the ultimate factor deemed essential to economic development regardless of future cost. Woinarski [165] cogently argues that before you start the killing, take the first step of understanding ultimate and proximate causation.

## 5. What Do the People Think through Attitudinal Surveys?

There have been many surveys relating to kangaroo management. In tourism, the kangaroo image is strongly associated with Australia [166] and international tourists have an appetite to see kangaroo species in the wild [167]. Various surveys relate to the commercial kangaroo industry, such as the acceptability of meat products [168] and welfare considerations [169]. Three papers [170,171,172] relate more generally to attitudes about wildlife (and pest) management. The first surveyed 793 adult Australians and showed an acceptability for lethal control only among (white) farmers. The remaining groups, who identified as “animal rights activists” or “wildlife conservationists”, were unaccepting and orientated towards non-lethal control. In the second study with 811 adults surveyed, perceptions of whether an animal group/species (kangaroos, horses, dingoes, red foxes) was native or not and always, sometimes or never a pest were assessed. Recognition of kangaroos as native was highest (96%) and 53% considered them never a pest (8% always, 36% in some contexts). The third study compared attitudes of 881 Australian citizens and 1287 USA citizens. Australians were more accepting of lethal control than US citizens. This may reflect the long history of lethal control of first, native wildlife, and later introduced species that had run wild, with poisoning as an exemplar [173].

Despite the vastness of the Australian continent, most of the population (~90%) is urban, living in cities and large rural towns. If they prefer to holiday in Bali not Bathurst, then they have little interaction with kangaroo species. The various possible interfaces are the media (usually hyperbole about abundance and negative effects), an intrusion of a species into a peri-urban area or in some localities in a park or golf course [174], in a zoo or nature reserve or national park, or along a road as roadkill or the target for a collision. Some interfaces may generate a positive attitude [175], others such as roadkill a decidedly negative one if a collision results [176]. For a significant portion of the population (29.7% or 7.5 million people), their birthplace is not Australia. They are likely to be indifferent towards kangaroos and their management, as migration to Australia is not based on the attraction of the wildlife but issues of improvement of economic and social well-being. To muster support for kangaroo species from a highly urban and immigrant population is challenging.

## 6. Resolving the Conflict—Conclusions

From this review, several perils of being populous emerge. (1) There is hyperbole about a species’ abundance that augments from abundant to overabundant, and then super or hyper abundant. For example, Hampton and colleagues state “Professional consumptive use of terrestrial wildlife typically involves in situ killing of hyperabundant large herbivores, particularly ungulates and marsupials” (p. 5) [177]. If the value and market for products are modest, as with the commercial kangaroo industry, then there may be an incentive for both the operator (optimal effort, marketing) and the regulator (defray perceptions of harm) to maintain high abundance. This creates conflict over the level of control [178]. (2) The conservation status of the abundant species is of “Least Concern” which may be perceived as “No Concern”. Thus, harm to the species and mortality are considered inconsequential. Advocacy for less harm to the species is considered misdirected in the face of pressing conservation issues where other species are on the brink of extinction [179]. (3) The species is labelled as overabundant and needs clever humans to save it from harm to itself. The typical kangaroo example is that mortality in drought results in high suffering so reducing abundance through lethal (and humane) means results in fewer individuals suffering. Droughts may be inevitable but they are unpredictable, although species in the northern savannas of Australia experience seasonal drought in the wet/dry tropics. Species in mesic environments are more likely to suffer from floods than droughts. Given anthropogenic climate change has increased extreme weather (droughts, high-intensity cyclones, high rainfall) and its consequences (starvation and wildfire, habitat destruction, flooding), there is a certain arrogance to humans as the “micro-managers”. (4) Abundant species make convenient scapegoats for mismanagement (e.g., over-grazing and land degradation), especially if they are large and obvious like kangaroos. Dogma about causes and consequences of abundance becomes entrenched. Proximate and ultimate causes of conflict with the abundant species are conflated and not fully resolved.

**Figure 7:**
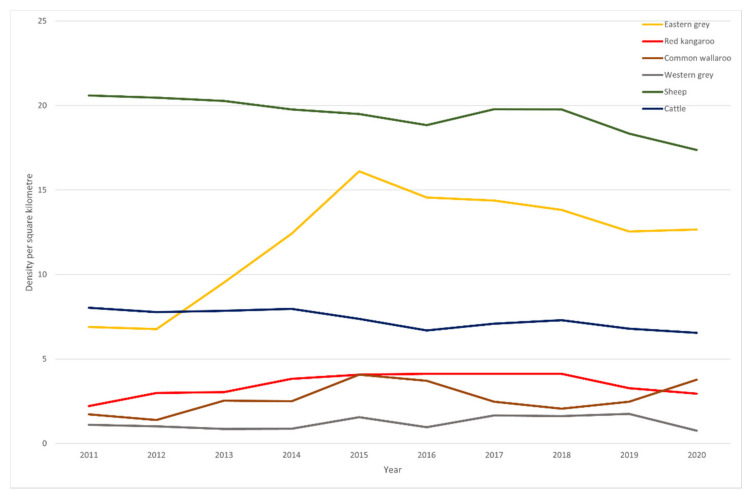
Estimates of the density of sheep, cattle, eastern grey kangaroos, red kangaroos, common wallaroos and western grey kangaroos in the combined grazing areas or survey areas of Queensland, New South Wales, South Australia and Western Australia from 2011–2020 (sources as for Figure 3 above).

The outlook for the six kangaroo species remaining widespread and unharmed is, in our opinion, poor. The trajectory over the last decade of the four species under regular population surveys may not support this pessimism (Figure 7). The eastern grey kangaroo seems to have prospered but this is not consistent across states, with a steep downward trend in Queensland [180]. The densities of other kangaroo species, and the livestock species, are relatively flat or trending down in the case of sheep. The decade has been complex with drought, extensive wildfire, extreme weather and a pandemic in the human population. These effects are not fully resolved in the kangaroo densities which are six months in arrears of the livestock censuses. Our cause for pessimism is the increased acceptance of harm to kangaroo species. There is an expansion of commercial harvesting zones in South Australia [181] and New South Wales [182]. In South Australia, the entire state, excluding metropolitan Adelaide and, significantly for our following proposal, the Alinytjara Wilura Aboriginal lands (for cultural reasons), is available for commercial kangaroo killing. In New South Wales, there has been liberalisation of accounting for harm and hunting through a general license (site-specific) to assist landholders with the control of native animals (farmer helper program). Licensed non-commercial killing of kangaroos exceeded commercial killing in South Australia (53%), but this was not replicated in other 2020 kangaroo harvest reports (New South Wales—23%, Queensland—16%, Western Australia—not reported). There is discontent with the ability of the commercial kangaroo harvest to control abundance and various other less-regulated strategies are being canvassed to reduce kangaroo species abundance [82].

Various consumptive and non-consumptive strategies have been promoted to manage and/or exploit abundant kangaroo species. These have attracted scientific and public interest but have subsequently languished. For example, Grigg [183] raised the prospect of improving the stability and biodiversity of the sheep rangelands through the consumption of kangaroo products whose profitability would partially or completely replace degradative sheep grazing. Thirty years on from that proposal he admits it had no traction. As an enterprise based on wool and/or meat from sheep fails, it is replaced by a mixed enterprise with cattle, and then a goat enterprise (or leapfrog from sheep to goats). With a volatile climate, pastoralists take an adaptive approach [184], but a divorce from traditional European style grazing to a night-time of shooting kangaroos is a leap too far.

Wilson and colleagues more recently have raised the prospect of a mixed consumptive livestock and kangaroo enterprise [136] but it is also likely to have no traction, especially if they promote it with the same sweeping generalisations and dogma that pervade the public face of kangaroo management [89]. They are right that we are likely to see more barbaric practices directed at suppression if not elimination of kangaroo species. We have already seen a replay of the battue (described in [65]) where kangaroos are driven and funnelled to a killing point [185,186]. The club was replaced with a lethal injection. Equally, we foresee a return of hunting with special breeds of dogs as described by Tench [56] at the founding of European Australia. However, horses will likely be replaced by an ATV (All Terrain Vehicle) or the killing will be performed directly by a person on an off-road motorcycle wielding a club. The former is a small side-step from feral pig hunting in Australia. However, it is curious that there is a chorus that conservation should be dispassionate not compassionate [18] given that in human society a lack of compassion is a sign of psychopathy, and is often associated with animal cruelty [187]. We should question and challenge acts of animal cruelty, not accept the least harmful choice of lethal control.

One of us [188] raised the prospect of a mixed enterprise with sheep and kangaroos with a more benign approach to the latter through wildlife tourism. Wildlife tourism with kangaroos gained some traction and spawned a number of studies in a sustainable tourism program (summarised in [167]), and research was conducted on the best ways to view kangaroos from a wildlife tourist’s perspective [189,190]. A resource was created, “The kangaroo trail map” (http://www.rootourism.com; accessed on 21 April 2021), which attracted no support from any state or territory tourism agency and now languishes as an essentially moribund website and a few boxes of (free) maps gathering dust.

Our recommended option is to allow Aboriginal Australia to re-emerge on the already secured IPAs (Indigenous Protected Areas) (https://www.niaa.gov.au/indigenous-affairs/environment/indigenous-protected-areas-ipas; accessed on 20 April 2021). These currently number 78 and cover nearly 750,000 km^2^. They contribute significant value to the national conservation estate among other lands under Aboriginal titles [191]. They are mostly remote in central, western and northern Australia. These regions have some issues of feral grazing animals but, in the north, macropodid communities remain intact. They provide “on country” employment through ranger programs [192] and could diversify into nature and culture-based tourism that do not preclude the consumption of local wildlife. Tourism infrastructure will be limited [193] and the expectation would be a boutique not a mass tourism experience. There are many examples of such programs in southern Africa, such as the conservancies in Namibia. The risk is that Aboriginal communities are seduced into the “real” economy (see [194]) of commercial kangaroo killing. This would seem like a perverse outcome of raiding the local larder to earn cash to buy a steak from the supermarket. There are also the seductions of cattle ranching, even though the northern industry was built on the exploitation of Aboriginal people employed with no or very low wages [195]. They may also be captive to mining, gas and petroleum interests. Regardless of risk, there is an appetite for conservation, re-wildling and managing landscapes in a more productive way for kangaroos [39,196]. Funding will be dependent on government largess, but given the ubiquity of kangaroo symbols in advertising and sporting teams, a levy could be placed on use with an existing UNDP conservation mission, the lion’s share fund (http://www.thelionsharefund.com; accessed on 21 April 2021).

In this scenario, some of the human–human conflict may be resolved. Grigg gets his livestock-free rangeland (albeit a northern savanna); Wilson, Edwards and Read get their mixed enterprise if the IPA managers collaborate with Aboriginal-owned pastoral properties (of which there are several); Croft gets his world-class wildlife experience; and the scene is completed with dingoes howling at night. The Aboriginal people get due recognition of their ongoing contribution to the well-being of the continent. We appear ready to embrace Aboriginal burning practices following the 2019–2020 “black summer” bushfires [197], which drew international attention to the consequences of anthropogenic climate change, and so this is a small step further.

The alternative is the further displacement of kangaroo species from most of Australia and their isolation in unviable economic zones. At this point, the kangaroo on the Australian coat of arms can be replaced with a bull, and the emu with a free-range chook (an Australian colloquialism for chicken). The European Australia will be complete, and the non-Aboriginal populace can declare “job done!”.

## Figures and Tables

**Table 1:** Stocking rate equivalents (dry sheep equivalent) of sheep, beef cattle and two species of kangaroo.

Livestock/Species	Dry Sheep Equivalent
Dry sheep ^1^	1.0
Breeding ewe ^1^	1.5
Dry cow 350–450 kg ^1^	8–10
Cow with calf ^1^	13–16
Bull ^1^	16
Red kangaroo (25 kg) ^2^	0.35
Western grey kangaroo (25 kg) ^3^	0.31

Source: ^1^ https://www.landscape.sa.gov.au/mr/publications/grazing-livestock-mlr-stocking-rates (accessed on 19 April 2021), ^2^ [87], ^3^ [88].

## Data Availability

The amalgamated data presented in this study are available within the article. The individual data are publicly available from the cited websites maintained by the Government of Australia.

## References

[1] Eldridge M.D.B., Beck R.M.D., Croft D.A., Travouillon K.J., Fox B.J. (2019). An emerging consensus in the evolution, phylogeny, and systematics of marsupials and their fossil relatives (Metatheria). J. Mammal..

[2] Hershkovitz I., Weber G.W., Quam R., Duval M., Grün R., Kinsley L., Ayalon A., Bar-Matthews M., Valladas H., Mercier N. (2018). The earliest modern humans outside Africa. Science.

[3] Rabett R.J. (2018). The success of failed Homo sapiens dispersals out of Africa and into Asia. Nat. Ecol. Evol..

[4] O’Connell J.F., Allen J., Williams M.A.J., Williams A.N., Turney C.S.M., Spooner N.A., Kamminga J., Brown G., Cooper A. (2018). When did *Homo sapiens* first reach Southeast Asia and Sahul?. Proc. Natl. Acad. Sci. USA.

[5] Field J., Fillios M., Wroe S. (2008). Chronological overlap between humans and megafauna in Sahul (Pleistocene Australia–New Guinea): A review of the evidence. Earth-Sci. Rev..

[6] Dawson T.J. (2012). Kangaroos—Biology of the Largest Marsupials.

[7] Lunney D., Purcell B., McLeod S., Grigg G., Pople T., Wolter S. (2018). Four decades of research and monitoring the populations of kangaroos in New South Wales: One of the best long-term datasets in Australia. Aust. Zool..

[8] Ritchie E.G., Martin J.K., Krockenberger A.K., Garnett S., Johnson C.N. (2008). Large-herbivore distribution and abundance: Intra- and interspecific niche variation in the tropics. Ecol. Monogr..

[9] Denny M., Lunney D., Hand S., Reed P., Butcher D. (1994). Investigating the past: An approach to detrmining the changes in the fauna of the Western Division of New South Wales since the first explorers. Future of the Fauna of Western New South Wales.

[10] Croft D.B., Wilson M., Croft D.B. (2005). The future of kangaroos—Going, going, Gone?. Kangaroos: Myths and Realities.

[11] Lele S. (2020). From wildlife-ism to ecosystem-service-ism to a broader environmentalism. Environ. Conserv..

[12] Young M.D., Gibbs M., Holmes W.E., Mills D.M.D., Harrington G.N., Wilson A.D., Young M.D. (1984). Socio-economic influences on pastoral management. Management of Australia’s Rangelands.

[13] Hacker R.B., Sinclair K., Pahl L. (2019). Prospects for ecologically and socially sustainable management of total grazing pressure in the southern rangelands of Australia. Rangel. J..

[14] Mills C.H., Waudby H., Finlayson G., Parker D., Cameron M., Letnic M. (2020). Grazing by over-abundant native herbivores jeopardizes conservation goals in semi-arid reserves. Glob. Ecol. Conserv..

[15] Bhatia S., Redpath S.M., Suryawanshi K., Mishra C. (2019). Beyond conflict: Exploring the spectrum of human–wildlife interactions and their underlying mechanisms. Oryx.

[16] Redpath S.M., Bhatia S., Young J. (2014). Tilting at wildlife: Reconsidering human–wildlife conflict. Oryx.

[17] Ramp D. (2013). Bringing compassion to the ethical dilemma in killing kangaroos for conservation: Comment on “Conservation through sustainable use” by Rob Irvine. J. Bioethical Inq..

[18] Hayward M.W., Callen A., Allen B.L., Ballard G., Broekhuis F., Bugir C., Clarke R.H., Clulow J., Clulow S., Daltry J.C. (2019). Deconstructing compassionate conservation. Conserv. Biol..

[19] Wallach A.D., Bekoff M., Batavia C., Nelson M.P., Ramp D. (2018). Summoning compassion to address the challenges of conservation. Conserv. Biol..

[20] Richardson H. (2012). Australia’s Amazing Kangaroos: Their Conservation, Unique Biology and Coexistence with Humans.

[21] Kingsford R.T., West R.S., Pedler R.D., Keith D.A., Moseby K.E., Read J.L., Letnic M., Leggett K.E.A., Ryall S.R. (2020). Strategic adaptive management planning—Restoring a desert ecosystem by managing introduced species and native herbivores and reintroducing mammals. Conserv. Sci. Pract..

[22] Croft D.B., Wilson M., Croft D.B. (2005). Kangaroos maligned—16 million years of evolution and two centuries of persecution. Kangaroos: Myths and Realities.

[23] Witte I., Wilson M., Croft D.B. (2005). Kangaroos—Misunderstood and maligned reproductive miracle workers. Kangaroos: Myths and Realities.

[24] Rolls E.C. (1984). They All Ran Wild: The Animals and Plants That Plague Australia.

[25] Gammage B. (2011). The Biggest Estate on Earth: How Aborigines Made Australia.

[26] Fletcher M.S., Hall T., Alexandra A.N. (2020). The loss of an indigenous constructed landscape following British invasion of Australia: An insight into the deep human imprint on the Australian landscape. Ambio.

[27] Edwards G.P., Allan G.E., Brock C., Duguid A., Gabrys K., Vaarzon-Morel P. (2008). Fire and its management in Central Australia. Rangel. J..

[28] Preece N.D. (2013). Tangible evidence of historic Australian indigenous savanna management. Austral Ecol..

[29] Russell-Smith J., Whitehead P., Cooke P. (2009). Culture, Ecology and Economy of Fire Management in North Australian Savannas: Rekindling the Wurrk Tradition.

[30] Jones R. (2012). Fire-Stick Farming. Fire Ecol..

[31] Pascoe B. (2018). Dark Emu.

[32] Denny M.J.S., Lavery H.J. (1985). The red kangaroo and the arid environment. The Kangaroo Keepers.

[33] Southwell C.J., Jarman P.J. (1987). Macropod studies at Wallaby Creek III. The effect of fire on pasture utilisation by macropodids and cattle. Aust. Wildl. Res..

[34] Gould R.A. (1971). Uses and effects of fire among the Western Desert Aborigines of Australia. Mankind.

[35] Caughley G., Brown B., Noble J. (1985). Movement of kangaroos after a fire in Mallee woodland. Aust. Wildl. Res..

[36] Yibarbuk D., Whitehead P.J., Russell-Smith J., Jackson D., Godjuwa C., Fisher A., Cooke P., Choquenot D., Bowman D.M.J.S. (2001). Fire ecology and Aboriginal land management in central Arnhem Land, northern Australia: A tradition of ecosystem management. J. Biogeogr..

[37] Murphy B.P., Bowman D.M.J.S. (2007). The interdependence of fire, grass, kangaroos and Australian Aborigines: A case study from central Arnhem Land, northern Australia. J. Biogeogr..

[38] Bowman D.M.J.S., Garde M., Saulwick A., Anderson A., Lilley I., O’Connor S. (2001). Fire is for hunting kangaroos: Interpreting Aboriginal accounts of landscape burning in central Arnhem Land. Histories of Old Ages: Essays in Honour of Rhys Jones.

[39] Codding B.F., Bliege Bird R., Kauhanen P.G., Bird D.W. (2014). Conservation or Co-evolution? Intermediate Levels of Aboriginal Burning and Hunting Have Positive Effects on Kangaroo Populations in Western Australia. Hum. Ecol..

[40] Altman J.C. (1984). The dietary utilisation of flora and fauna by contemporary hunter-gatherers at Momega outstation, north-central Arnhem Land. Aust. Aborig. Stud..

[41] O’Connell J.F. (1980). Notes on the manufacture and use of a kangaroo skin waterbag. Aust. Inst. Aborig. Stud. Newsl..

[42] Meagher S.J., Ride W.D.L., Berndt R.M., Berndt C.H. (1978). Use of natural resources by the Aborigines of south-western Australia. Aborigines in the West: Their Past and Their Present.

[43] Mills V. (2021). Kangaroo-Bone Tools Found in Riwi Cave in the Kimberley Are Thought to be 35,000 Years Old.

[44] Finch D., Gleadow A., Hergt J., Ourzman S. (2021). This 17,500-Year-Old Kangaroo in the Kimberley Is Australia’s Oldest Aboriginal Rock Painting. The Conversation.

[45] Newsome A.E. (1980). The eco-mythology of the red kangaroo in central Australia. Mankind.

[46] Telfer W.R., Garde M.J. (2006). Indigenous knowledge of rock kangaroo ecology in Western Arnhem Land, Australia. Hum. Ecol..

[47] Liebenberg L. (1990). The Art of Tracking: The Origin of Science.

[48] Flannery T.F. (1994). The Future Eaters: An Ecological History of the Australasian Lands and People.

[49] Saltre F., Rodriguez-Rey M., Brook B.W., Johnson C.N., Turney C.S., Alroy J., Cooper A., Beeton N., Bird M.I., Fordham D.A. (2016). Climate change not to blame for late Quaternary megafauna extinctions in Australia. Nat. Commun..

[50] Wroe S., Field J., Fullagar R., Jermin L.S. (2004). Megafaunal extinction in the late Quaternary and the global overkill hypothesis. Alcheringa.

[51] O’Connell J.F., Allen J. (2012). The restaurant at the end of the Universe: Modelling the colonisation of Sahul. Aust. Archaeol..

[52] Prowse T.A.A., Johnson C.N., Bradshaw C.J.A., Brook B.W. (2014). An ecological regime shift resulting from disrupted predator-prey interactions in Holocene Australia. Ecology.

[53] Roberts R.G. (2014). A pardon for the dingo. Science.

[54] Gascoinge J. (2020). From Captain Cook to the First Fleet: How Botany Bay Was Chosen over Africa as a New British Penal Colony.

[55] Smyth A.B. (1979). The Journal of Arthur Bowes Smyth. Surgeon, Lady Penrhyn, 1787–1789.

[56] Tench W.A. (1961). Sydney’s First Four Years.

[57] Oxley J. (1820). Journals of Two Expeditions into the Interior of New South Wales.

[58] Newsome A.E. (1975). An ecological comparison of the two arid-zone kangaroos of Australia, and their anomalous prosperity since the introduction of ruminant stock to their environment. Q. Rev. Biol..

[59] Woinarski J.C., Burbidge A.A., Harrison P.L. (2015). Ongoing unraveling of a continental fauna: Decline and extinction of Australian mammals since European settlement. Proc. Natl. Acad. Sci. USA.

[60] Lindenmayer D.B. (2015). Continental-level biodiversity collapse. Proc. Natl. Acad. Sci. USA.

[61] Ben-Ami D., Ramp D. (2006). The effect of road-based fatalities on the viability of a peri-urban swamp wallaby population. J. Wildl. Manag..

[62] Cooke B.D. (2020). Swamp wallaby (*Wallabia bicolor*) distribution has dramatically increased following sustained biological control of rabbits. Aust. Mammal..

[63] Johnson C.N., Smith B. (2015). An ecological view of the dingo. The Dingo Debate: Origins, Behaviour and Conservation.

[64] Frith H.J., Calaby J.H. (1969). Kangaroos.

[65] Hornadge B. (1972). If It Moves, Shoot It: A Squint at Some Australian Attitudes towards the Kangaroo.

[66] Silcock J.L., Piddocke T.P., Fensham R.J. (2013). Illuminating the dawn of pastoralism: Evaluating the record of European explorers to inform landscape change. Biol. Conserv..

[67] Auty J. (2004). Red plague grey plague: The kangaroo myths and legends. Aust. Mammal..

[68] Denny M.J.S. (1982). Kangaroos: An historical perspective. Parks and Wildlife Kangaroos.

[69] Fillios M., Gordon C., Koch F., Letnic M. (2010). The effect of a top predator on kangaroo abundance in arid Australia and its implications for archaeological faunal assemblages. J. Archaeol. Sci..

[70] Neville-Rolfe C.W., Morris E.E. (1889). Some birds and beasts. Cassell’s Picturesque Australasia.

[71] Morris E.E. (1978). Australia’s First Century, 1788–1888.

[72] Saville-Kent W. (1897). The Naturalist in Australia.

[73] Montgomery J. (1969). Is it too late to save the Big Red?. Animals.

[74] Kirkpatrick J.B., Amos P.J., Lavery H.J. (1985). The kangaroo industry. The Kangaroo Keepers.

[75] Lunney D. (2010). A history of the debate (1948–2009) on the commercial harvesting of kangaroos, with particular reference to New South Wales and the role of Gordon Grigg. Aust. Zool..

[76] Edwards G.P., Croft D.B., Dawson T.J. (1994). Observations of differential sex/age class mobility in red kangaroos (*Macropus rufus*). J. Arid Environ..

[77] Herbert C.A. (2004). Long-acting contraceptives: A new tool to manage overabundant kangaroo populations in nature reserves and urban areas. Aust. Mammal..

[78] Caughley G., Jewell P.A., Holt S., Hart D. (1981). Overpopulation. Problems in the Management of Locally Abundant Wild Animals.

[79] ACT Kangaroo Advisory Committee (1997). Living with Eastern Grey Kangaroos in the ACT—Public Land: Third Report to the Minister for the Environment, Land and Planning.

[80] Ben-Ami D., Mjadwesch R. (2017). Integrating animal protection criteria into conservation management: A case study of the management of Eastern Grey Kangaroos in the ACT. Isr. J. Ecol. Evol..

[81] Fletcher D., Lunney D., Eby P., Hutchings P., Burgin S. (2007). Managing Eastern Grey Kangaroos *Macropus giganteus* in the Australian Capital Territory: Reducing the overabundance—of opinion. Pest or Guest: The Zoology of Overabundance.

[82] The Kangaroo Management Taskforce (2020). Options for Integrated Kangaroo Management in the Western Region: A Practical Guide for Active Management.

[83] Hacker R.B., Sinclair K., Waters C.M. (2019). Total grazing pressure—A defining concept for extensive pastoral systems in the southern rangelands of Australia. Rangel. J..

[84] Hadlington P., Louise B., Staunton I. (2007). Australian Termites.

[85] Bergstrom D.M., Wienecke B.C., van den Hoff J., Hughes L., Lindenmayer D.B., Ainsworth T.D., Baker C.M., Bland L., Bowman D., Brooks S.T. (2021). Combating ecosystem collapse from the tropics to the Antarctic. Glob. Chang. Biol..

[86] Norbury G.L., Norbury D.C. (1993). The distribution of red kangaroos in relation to range regeneration. Rangel. J..

[87] Munn A.J., Dawson T.J., McLeod S.R., Croft D.B., Thompson M.B., Dickman C.R. (2008). Field metabolic rate and water turnover of red kangaroos and sheep in an arid rangeland: An empirically derived dry-sheep-equivalent for kangaroos. Aust. J. Zool..

[88] Munn A.J., Kalkman L., Skeers P., Roberts J.A., Bailey J., Dawson T.J. (2016). Field metabolic rate, movement distance, and grazing pressures by western grey kangaroos (*Macropus fuliginosus melanops*) and Merino sheep (*Ovis aries*) in semi-arid Australia. Mamm. Biol..

[89] Wilson G.R., Read J. (2021). A US ban on kangaroo leather would be an animal welfare disaster—and a missed farming opportunity. The Conversation.

[90] Williams A.N. (2013). A new population curve for prehistoric Australia. Proc. R. Soc. B.

[91] Bradshaw C.J.A., Norman K., Ulm S., Williams A.N., Clarkson C., Chadœuf J., Lin S.C., Jacobs Z., Roberts R.G., Bird M.I. (2021). Stochastic models support rapid peopling of Late Pleistocene Sahul. Nat. Commun..

[92] Wilson G.R. Co-production of livestock and kangaroos: A review of impediments and opportunities to collaborative regional management of wildlife resources. Proceedings of the Conservation through Sustainable Use of Wildlife Conference.

[93] Lonsdale W.M. (1994). Inviting trouble: Introduced pasture species in northern Australia. Aust. J. Ecol..

[94] Ramp D., Caldwell J., Edwards K., Warton D., Croft D.B. (2005). Modelling of wildlife fatality hotspots along the Snowy Mountains highway in New South Wales, Australia. Biol. Conserv..

[95] Edwards G.P., Croft D.B., Dawson T.J. (1996). Competition between red kangaroos (*Macropus rufus*) and sheep (*Ovis aries*) in the arid rangelands of Australia. Aust. J. Ecol..

[96] Hooper P. (1999). Kangaroo blindness and some other new viral diseases in Australia. Aust. Vet. J..

[97] Quesnel L., King W.J., Coulson G., Festa-Bianchet M. (2018). Tall young females get ahead: Size-specific fecundity in wild kangaroos suggests a steep trade-off with growth. Oecologia.

[98] Menz C.S., Carter A.J., Best E.C., Freeman N.J., Dwyer R.G., Blomberg S.P., Goldizen A.W. (2020). Higher sociability leads to lower reproductive success in female kangaroos. R. Soc. Open Sci..

[99] Banks P., Newsome A.E., Dickman C.R. (2000). Predation by red foxes limits recruitment in populations of eastern grey kangaroos. Austral Ecol..

[100] Letnic M., Ritchie E.G., Dickman C.R. (2012). Top predators as biodiversity regulators: The dingo *Canis lupus dingo* as a case study. Biol. Rev..

[101] Allen B.L., Fleming P.J.S., Allen L.R., Engeman R.M., Ballard G., Leung L.K.P. (2013). As clear as mud: A critical review of evidence for the ecological roles of Australian dingoes. Biol. Conserv..

[102] Caughley G., Grigg G.C., Caughley J., Hill G.J.E. (1980). Does dingo predation control the densities of kangaroos and emus?. Aust. Wildl. Res..

[103] Newsome A.E. (1990). The control of vertebrate pests by vertebrate predators. Trends Ecol. Evol..

[104] Newsome A.E., Catling P.C., Cooke B.D., Smyth R. (2001). Two ecological universes separated by the dingo barrier fence in semi-arid Australia: Interactions between landscapes, herbivory and carnivory, with and without dingoes. Rangel. J..

[105] Fisher A.G., Mills C.H., Lyons M., Cornwell W.K., Letnic M. (2021). Remote sensing of trophic cascades: Multi-temporal landsat imagery reveals vegetation change driven by the removal of an apex predator. Landsc. Ecol..

[106] Morris T., Letnic M. (2017). Removal of an apex predator initiates a trophic cascade that extends from herbivores to vegetation and the soil nutrient pool. Proc. R. Soc. B.

[107] Croft D.B., Montague-Drake R., Dowle M., Dickman C.R., Lunney D., Burgin S. (2007). Biodiversity and water point closure: Is the grazing piosphere a persistent effect?. Animals of Arid Australia: Out There on Their Own?.

[108] Thomson P.C. (1992). The behavioural ecology of dingoes in north-western Australia. 3. Hunting and feeding behaviour, and diet. Wildl. Res..

[109] Shepherd N.C. (1981). Predation of red kangaroos, *Macropus rufus*, by the dingo, *Canis familiaris dingo* (Blumenbach), in north-western New South Wales. Aust. Wildl. Res..

[110] Fleming P., Corbett L., Harden R.H., Thomson P.C. (2001). Managing the Impacts of Dingoes and Other Wild Dogs.

[111] Brunton E.A., Srivastava S.K., Burnett S. (2018). Spatial ecology of an urban eastern grey kangaroo (*Macropus giganteus*) population: Local decline driven by kangaroo–vehicle collisions. Wildl. Res..

[112] Brunton E.A., Srivastava S.K., Schoeman D.S., Burnett S. (2018). Quantifying trends and predictors of decline in eastern grey kangaroo (*Macropus giganteus*) populations in a rapidly urbanising landscape. Pac. Conserv. Biol..

[113] Arnold G.W., Weeldenburg J.R., Ng V.M. (1995). Factors affecting the distribution and abundance of western grey kangaroos (*Macropus fuliginosus*) and euros (*M. robustus*) in a fragmented landscape. Landsc. Ecol..

[114] Short J., Grigg G.C. (1982). The abundance of kangaroos in suboptimal habitats: Wheat, intensive pastoral, and mallee. Aust. Wildl. Res..

[115] Freudenberger D., Hodgkinson K., Noble J., Ludwig J., Tongway D., Freudenberger D., Noble J., Hodgkinson K. (1997). Causes and consequences of landscape dysfunction in rangelands. Landscape Ecology—Function and Management.

[116] Driscoll D.A., Catford J.A., Barney J.N., Hulme P.E., Inderjit, Martin T.G., Pauchard A., Pysek P., Richardson D.M., Riley S. (2014). New pasture plants intensify invasive species risk. Proc. Natl. Acad. Sci. USA.

[117] Kean L., Price M.O. (2002). The extent of Mission grasses and Gamba grass in the Darwin region of Australia’s Northern Territory. Pac. Conserv. Biol..

[118] Rossiter-Rachor N.A., Setterfield S.A., Douglas M.M., Hutley L.B., Cook G.D. (2008). *Andropogon gayanus* (Gamba Grass) invasion increases fire-mediated nitrogen losses in the tropical savannas of Northern Australia. Ecosystems.

[119] Bowman D. (2012). Bringing elephants to Australia?. Nature.

[120] Fisher D.O., Blomberg S.P., Owens I.P. (2003). Extrinsic versus intrinsic factors in the decline and extinction of Australian marsupials. Proc. R. Soc. B.

[121] Caughley G., Shepherd N., Short J. (1987). Kangaroos: Their Ecology and Management in the Sheep Rangelands of Australia.

[122] Edwards G.P., Dawson T.J., Croft D.B. (1995). The dietary overlap between red kangaroos (*Macropus rufus*) and sheep (*Ovis aries*) in the arid rangelands of Australia. Aust. J. Ecol..

[123] Witte I. (2002). Spatio-Temporal Interations of Mammalian Herbivores in the Arid Zone. Ph.D. Thesis.

[124] Norbury G.L., Norbury D.C., Hacker R.B. (1993). Impact of red kangaroos on the pasture layer in the Western Australian arid zone. Rangel. J..

[125] Tyndale-Biscoe H. (2005). Kangaroos and sheep: The unequal contest. Australas. Sci..

[126] Newsome A.E. (1965). The distribution of red kangaroos, *Megaleia rufa* (Desmarest), about sources of persistent food and water in central Australia. Aust. J. Zool..

[127] Newsome A.E. (1971). Competition between wildlife and domestic stock. Aust. Vet. J..

[128] Frank A.S.K., Wardle G.M., Greenville A.C., Dickman C.R. (2016). Cattle removal in arid Australia benefits kangaroos in high quality habitat but does not affect camels. Rangel. J..

[129] Reid A.M., Murphy B.P., Vigilante T., Barry L.A., Bowman D.M.J.S. (2020). Carbon isotope analysis shows introduced bovines have broader dietary range than the largest native herbivores in an Australian tropical savanna. Austral Ecol..

[130] Reid A.M., Murphy B.P., Vigilante T., Bowman D.M.J.S. (2020). Distribution and abundance of large herbivores in a northern Australian tropical savanna: A multi-scale approach. Austral Ecol..

[131] Ritchie E.G., Martin J.K., Johnson C.N., Fox B.J. (2009). Separating the influences of environment and species interactions on patterns of distribution and abundance: Competition between large herbivores. J. Anim. Ecol..

[132] Ealey E.H.M. (1967). Ecology of the euro, *Macropus robustus* (Gould), in north-western Australia—II. Behaviour, movements, and drinking patterns. CSIRO Wildl. Res..

[133] Ealey E.H.M. (1967). Ecology of the euro, Macropus robustus (Gould), in north-western Australia—I. The environment and changes in euro and sheep populations. CSIRO Wildl. Res..

[134] Ealey E.H.M., Main A.R. (1967). Ecology of the euro, *Macropus robustus* (Gould), in north-western Australia—III. Seasonal changes in nutrition. CSIRO Wildl. Res..

[135] Hume I.D. (1999). Marsupial Nutrition.

[136] Wilson G.R., Edwards M. (2019). Professional kangaroo population control leads to better animal welfare, conservation outcomes and avoids waste. Aust. Zool..

[137] Croft D.B. (1985). Inter- and intraspecific conflict between arid-zone kangaroos at watering points. Aust. Wildl. Res..

[138] Fanning P. (1994). Long-term contemporary erosion rates in arid rangelands environments in western New South Wales, Australia. J. Arid Environ..

[139] Condon R.W. (2002). Out of the West: A Historical Perspective of the Western Division of New South Wales.

[140] Montague-Drake R., Croft D.B. (2004). Do kangaroos exhibit water-focused grazing patterns in arid New South Wales? A case study in Sturt National Park. Aust. Mammal..

[141] Letnic M., Crowther M.S. (2013). Patterns in the abundance of kangaroo populations in arid Australia are consistent with the exploitation ecosystems hypothesis. Oikos.

[142] Lavery T.H., Pople A.R., McCallum H.I. (2018). Going the distance on kangaroos and water: A review and test of artificial water point closures in Australia. J. Arid Environ..

[143] Harris D.B., Gregory S.D., Brook B.W., Ritchie E.G., Croft D.B., Coulson G., Fordham D.A. (2014). The influence of non-climate predictors at local and landscape resolutions depends on the autecology of the species. Austral Ecol..

[144] Koerner S.E., Smith M.D., Burkepile D.E., Hanan N.P., Avolio M.L., Collins S.L., Knapp A.K., Lemoine N.P., Forrestel E.J., Eby S. (2018). Change in dominance determines herbivore effects on plant biodiversity. Nat. Ecol. Evol..

[145] Van Langvelde F., van de Claudius A.D.M., Kumar L., van de Koppel J., de Ridder N., van Andel J., Skidmore A.K., Hearne J.W., Stroosnujder L., Bond W.W. (2003). Effects of fire and herbivory on the stability of savanna ecosystems. Ecology.

[146] Croft D.B., Croft D.B., Ganslosser U. (1996). Locomotion, foraging competition and group size. Comparison of Marsupial and Placental Behaviour.

[147] Dawson T.J., Ellis B.A. (1994). Diets of mammalian herbivores in Australian arid shrublands: Seasonal effects on overlap between red kangaroos, sheep and rabbits and on dietary niche breadths and electivities. J. Arid Environ..

[148] Dawson T.J., Ellis B.A. (1996). Diets of mammalian herbivores in Australian arid, hilly shrublands: Seasonal effects on overlap between euros (hill kangaroos), sheep and feral goats, and on dietary niche breadths and electivities. J. Arid Environ..

[149] Wann J.M., Bell D.T. (1997). Dietary preferences of the black-gloved wallaby (*Macropus irma*) and the western grey kangaroo (*M. fuliginosus*) in Whiteman Park, Perth, Western Australia. J. R. Soc. West. Aust..

[150] Eldridge D.J., Rath D. (2002). Hip holes: Kangaroo (*Macropus* spp.) resting sites modify the physical and chemical environment of woodland soils. Austral Ecol..

[151] Eldridge D.J., Ding J., Travers S.K. (2021). Low-intensity kangaroo grazing has largely benign effects on soil health. Ecol. Manag. Restor..

[152] Iles J., Kellaway J., Kobayashi T., Mazumber D., Knowles L., Priddel D., Saintilan N. (2010). Grazing kangaroos as local recyclers of energy on semiarid floodplains. Aust. J. Zool..

[153] Jarman P.J. (1994). The eating of seedheads by species of Macropodidae. Aust. Mammal..

[154] Martine C.T., Boni A.J., Capaldi E.A., Lionheart G.E., JordonThaden I.E. (2016). Evidence of rock kangaroo seed dispersal via faecal seed storage in a tropical monsoon community. North. Territ. Nat..

[155] Rees J.D., Kingsford R.T., Letnic M. (2017). In the absence of an apex predator, irruptive herbivores suppress grass seed production: Implications for small granivores. Biol. Conserv..

[156] Prowse T.A.A., O’Connor P.J., Collard S.J., Rogers D.J. (2019). Eating away at protected areas: Total grazing pressure is undermining public land conservation. Glob. Ecol. Conserv..

[157] Howland B.W.A., Driscoll D.A. (2018). At high densities kangaroo grazing can reduce biodiversity. Austral Ecol..

[158] Mawson P.R., Hampton J.O., Dooley B. (2016). Subsidized commercial harvesting for cost-effective wildlife management in urban areas: A case study with kangaroo sharpshooting. Wildl. Soc. Bull..

[159] Department of Environment and Water (2020). 2019 Harvest Report: Commercial Kangaroo Harvest, South Australia.

[160] Snape M., Caley P., Baines G., Fletcher D. (2018). Kangaroos and Conservation: Assessing the Effects of Kangaroo Grazing in Lowland Grassy Ecosystems.

[161] Higginbottom K.B., Page S., Coulson G., Eldridge M.D.B. (2008). Monitoring the fate of translocated eastern grey kangaroos at the Gold Coast. Macropods: The Biology of Kangaroos, Wallabies and Rat-Kangaroos.

[162] Cowan M., Blythman M., Angus J., Gibson L. (2020). Post-Release Monitoring of Western Grey Kangaroos (*Macropus fuliginosus*) Relocated from an Urban Development Site. Animals.

[163] Poiani A., Coulson G., Salamon D., Holland S., Nave C.D. (2002). Fertility control of eastern grey kangaroos: Do levonorgestrel implants affect behavior?. J. Wildl. Manag..

[164] Lebbink G., Dwyer J.M., Fensham R.J. (2021). Managed livestock grazing for conservation outcomes in a Queensland fragmented landscape. Ecol. Manag. Restor..

[165] Woinarski J.C.Z. (2019). Killing Peter to save Paul: An ethical and ecological basis for evaluating whether a native species should be culled for the conservation benefit of another native species. Aust. Zool..

[166] Hill B., Arthurson T., Challio L. (2002). Kangaroos in the Marketing of Australia: Potentials and Practice.

[167] Higginbottom K.B., Northrope C.L., Croft D.B., Hill B., Fredline E. (2004). The role of kangaroos in Australian tourism. Aust. Mammal..

[168] Ampt P., Owen K. (2008). Consumer Attitudes to Kangaroo Meat Products.

[169] Sharp T.M., McLeod S.R. (2020). The Development of a New Code of Practice for the Commercial Harvesting of Kangaroos.

[170] Van Eeden L.M., Newsome T.M., Crowther M.S., Dickman C.R., Bruskotter J. (2019). Social identity shapes support for management of wildlife and pests. Biol. Conserv..

[171] Van Eeden L.M., Newsome T.M., Crowther M.S., Dickman C.R., Bruskotter J. (2020). Diverse public perceptions of species’ status and management align with conflicting conservation frameworks. Biol. Conserv..

[172] Van Eeden L.M., Slagle K., Newsome T.M., Crowther M.S., Dickman C.R., Bruskotter J.T. (2020). Exploring nationality and social identity to explain attitudes toward conservation actions in the United States and Australia. Conserv. Biol..

[173] Philip J. (2019). The Institutionalisation of Poison: A historical review of vertebrate pest control in Australia, 1814 to 2018. Aust. Zool..

[174] Coulson G., Cripps J.K., Wilson M.E. (2014). Hopping Down the Main Street: Eastern Grey Kangaroos at Home in an Urban Matrix. Animals.

[175] Croft D.B., Wilson M. (1999). Rangeland Kangaroos—A world class wildlife experience. Kangaroos Betrayed: World’s Largest Wildlife Slaughter.

[176] Ang J.Y., Gabbe B., Cameron P., Beck B. (2019). Animal-vehicle collisions in Victoria, Australia: An under-recognised cause of road traffic crashes. Emerg. Med. Australas..

[177] Hampton J.O., Hyndman T.H., Allen B.L., Fischer B. (2021). Animal Harms and Food Production: Informing Ethical Choices. Animals.

[178] McLeod S.R., Hacker R.B. (2019). Balancing stakeholder interests in kangaroo management—Historical perspectives and future prospects. Rangel. J..

[179] Johnson C., Woinarski J., Cooney R. (2015). Bans on kangaroo products are a case of emotion trumping science. The Conversation.

[180] Environmental Services and Regulation, Department of Environment and Science (2021). Queensland Commercial Macropod Management Plan: Annual Report 2020.

[181] Department of Environment and Resources (2010). 2011 Kangaroo Harvest Quota Report for South Australia.

[182] Department of Planning Industry & Environment (2021). 2020 Annual Report: New South Wales Commercial Kangaroo Harvest Management Plan 2017–2021.

[183] Grigg G. (2017). Eating kangaroo (good) and goat (bad) for rangelands. Aust. Zool..

[184] Berry E., Metternicht G., Baumber A. (2019). ‘This country just hangs tight’: Perspectives on managing land degradation and climate change in far west NSW. Rangel. J..

[185] Colgan S.A., Perkins N.R., Green L.A. (2019). The large-scale capture of eastern grey kangaroos (*Macropus giganteus*) and red kangaroos (*Osphranter rufus*) and its application to a population management project. Aust. Vet. J..

[186] Ward T. (2009). Fences, boundaries, and jurisdictions: Canberra’s kangaroo ‘cull’ and the law. Aust. Anim. Prot. Law, J..

[187] Rock R.C., Haugh S., Davis K.C., Anderson J.L., Johnson A.K., Jones M.A., Salekin R.T. (2021). Predicting animal abuse behaviors with externalizing and psychopathic personality traits. Personal. Individ. Differ..

[188] Croft D.B. (2000). Sustainable use of wildlife in western New South Wales: Possibilities and problems. Rangel. J..

[189] Wolf I.D., Croft D.B. (2010). Minimizing disturbance to wildlife by tourists approaching on foot or in a car: A study of kangaroos in the Australian rangelands. Appl. Anim. Behav. Sci..

[190] Wolf I.D., Croft D.B. (2012). Observation techniques that minimize impacts on wildlife and maximize visitor satisfaction in night-time tours. Tour. Manag. Perspect..

[191] Renwick A.R., Robinson C.J., Garnett S.T., Leiper I., Possingham H.P., Carwardine J. (2017). Mapping Indigenous land management for threatened species conservation: An Australian case-study. PLoS ONE.

[192] Schultz R., Abbott T., Yamaguchi J., Cairney S. (2019). Australian Indigenous Land Management, Ecological Knowledge and Languages for Conservation. Ecohealth.

[193] Croft D.B., Leiper N. (2001). Assessment of Opportunities for Overseas Tourism Based on Wild Kangaroos.

[194] Kurmelovs R. (2021). ‘Like champagne, mate’: How a US kangaroo ban could kill off an Indigenous opportunity Indigenous Australians?. The Guardian.

[195] Ward C. (2016). A Handful of Sand: The Gurinji Struggle after the Walk-Off.

[196] Muhic J., Abbott E., Ward M.J. (2012). The warru (*Petrogale lateralis*) reintroduction project on the Anangu Pitjantjatjara Yankunytjatjara Lands, South Australia. Ecol. Manag. Restor..

[197] Steffersen V. (2020). Fire Country: How Indigenous Fire Management Could Help Save Australia.

